# Pharmacokinetic/Pharmacodynamic modelling of Saxagliptin and its active metabolite, 5-hydroxy Saxagliptin in rats with Type 2 Diabetes Mellitus

**DOI:** 10.1186/s40360-024-00757-3

**Published:** 2024-06-26

**Authors:** Tianyan Wang, Ting Tao, Yi Liu, Jie Dong, Shanhong Ni, Yun Liu, Yanli Li, Ning Xu, Zengxian Sun

**Affiliations:** 1 Department of Pharmacy, The Affiliated Lianyungang Hospital of Xuzhou Medical University, The First People’s Hospital of Lianyungang, Lianyungang, 222061 China; 2 Endocrinology Department, The Affiliated Lianyungang Hospital of Xuzhou Medical University, The First People’s Hospital of Lianyungang, Lianyungang, 222061 China

**Keywords:** Pharmacokinetic/Pharmacodynamic model, Saxagliptin, 5-Hydroxy Saxagliptin, Type 2 Diabetes Mellitus rats, DPP-4

## Abstract

**Background and purposes:**

It is unclear whether the parent Saxagliptin (SAX) in vivo is the same as that in vitro, which is twice that of 5-hydroxy Saxagliptin (5-OH SAX). This study is to construct a Pharmacokinetic-Pharmacodynamic (PK-PD) link model to evaluate the genuine relationship between the concentration of parent SAX in vivo and the effect.

**Methods:**

First, we established a reliable Ultra Performance Liquid Chromatography-Mass Spectrometry (UPLC-MS/MS) method and DPP-4 inhibition ratio determination method. Then, the T2DM rats were randomly divided into four groups, intravenous injection of 5-OH SAX (0.5 mg/kg) and saline group, intragastric administration of SAX (10 mg/kg) and Sodium carboxymethyl cellulose (CMC-Na) group. Plasma samples were collected at different time points for subsequent testing. Finally, we used the measured concentrations and inhibition ratios to construct a PK-PD link model for 5-OH SAX and parent SAX.

**Results:**

A two-compartment with additive model showed the pharmacokinetic process of SAX and 5-OH SAX, the concentration-effect relationship was represented by a sigmoidal E_max_ model and sigmoidal E_max_ with E_0_ model for SAX and 5-OH SAX, respectively. Fitting parameters showed SAX was rapidly absorbed after administration (*T*_max_=0.11 h, *t*_1/2, ka_=0.07 h), widely distributed in the body (V ≈ 20 L/kg), plasma exposure reached 3282.06 ng*h/mL, and the elimination half-life was 6.13 h. The maximum plasma dipeptidyl peptidase IV (DPP-4) inhibition ratio of parent SAX was 71.47%. According to the final fitting parameter EC_50_, EC_50, 5−OH SAX_=0.46EC_50, SAX(parent)_, it was believed that the inhibitory effect of 5-OH SAX was about half of the parent SAX, which is consistent with the literature.

**Conclusions:**

The PK-PD link model of the parent SAX established in this study can predict its pharmacokinetic process in T2DM rats and the strength of the inhibitory effect of DPP-4 based on non-clinical data.

**Supplementary Information:**

The online version contains supplementary material available at 10.1186/s40360-024-00757-3.

## Introduction

Diabetes mellitus (DM) is a type of metabolic disease caused by insulin dysfunction resulting from absolutely or relatively insufficient insulin secretion. According to International Diabetes Federation in 2021, there were 537 million diabetic patients worldwide [1]. Based on current rate of increase, the number of adults suffering from diabetes will reach 783 million in 2045 [1]. China holds the highest prevalence of diabetes in the world, and it is estimated that there will be 14.086 million diabetic patients in 2021 [2]. According to the guideline for Prevention and Treatment of Type 2 Diabetes mellitus (T2DM) in China in 2020 [3], the prevalence of diabetes among people aged 18 and over in China had reached 11.2% with more than 90% were T2DM, while the proportion of undiagnosed diabetes was as high as 54%, but lower than before (62% [4]).

At present, the medicines to treat T2DM were sodium-dependent glucose transporters 2 inhibitors, glucagon-like peptide-1 receptor agonists, DPP-4 inhibitors, Thiazolidinediones, Sulfony ureas (2nd generation) and human insulin or analogs [5]. The DPP-4 inhibitor is a novel oral hypoglycemic drugs newly marketed in recent years. It plays a hypoglycemic role by increasing the level of glucagon like peptide-1, which stimulates insulin secretion [6]. With the continuous development of technology, FDA approved Sitagliptin, SAX, Vildagliptin and Linagliptin, etc. for marketing [7]. Among them, SAX is a potent and selective DPP-4 inhibitor.

The bioavailability of oral 5 mg SAX was 75%, and about 50% of the dose of SAX was metabolized in the liver by Cytochrome P450 3A4/5, (CYP3A4/5) to the main metabolite 5-OH SAX [8]. Its systemic exposure in plasma was about 3 times that of the parent drug SAX [9], and its activity is about half of SAX [10]. As a substrate of CYP3A4/5, SAX may have drug interactions with CYP3A4/5 inhibitors or inducers. The liver and kidneys rapidly metabolize SAX, while 5-OH SAX is mainly metabolized in the kidneys. There is possibility that liver and kidney dysfunction may also affect the PK and PD of SAX and 5-OH SAX [9, 11–14].

PK/PD model can describe the PK and PD process of drugs in the body. According to that, we can predict the entire course of the drug in vivo under a certain dosage regimen and screen the optimal dose then, which has now played an increasingly important role in new drug development and clinical use.

The preclinical and clinical studies affirmed the SAX inhibitory effect on DPP-4 enzyme. However, few studies have removed the 5-OH SAX effect and only discussed the inhibitory effect of maternal SAX on DPP-4 enzyme in vivo. PK/PD modeling of the parent SAX is helpful to understand the true PK/PD course in vivo, which can be applied to estimate the dose or predict the strength of the SAX effect for some special patients (such as liver and kidney damage, drug interactions).

First, we established an accurate and reliable UPLC-MS/MS method to detect the plasma concentrations of SAX and 5-OH SAX of T2DM rats, which was used to create the PK model then. Gly-Pro-pNA was used to build up an enzyme activity determination method. Finally, we found the PK/PD model based on the plasma concentrations, DPP-4 inhibition ratios and time. According to the model, we described the genuine relationship between the concentration of parent SAX in vivo and the effect.

## Materials and methods

### Chemical and reagents

SAX and 5-OH SAX were received from Haosen Pharmaceutical Group Co., Ltd (Jiangsu, China). Glycyl-prolyl-p-nitroaniline (Gly-Pro-pNA), streptozocin and pNA were all obtain from Sigma-Aldrich (Saint Louis, MO, USA). Vildagliptin, Citric acid and sodium citrate were purchased from Aladdin (Hangzhou, China). The purity of these reagents all above was higher than 98%. Acetonitrile and methanol of high-performance liquid chromatography (HPLC) grade were purchased from Merck (Darmstadt, Germany).

### Animals and study design

Male Sprague-Dawley (SD) rats weighing 120–140 g were purchased from the Experimental Animal Center of Nantong University (SCXK (Su):2019-0001), China. The Institutional Animal Care and Use Committee (IACUC) approved the animal experimental protocols. The rats were maintained under standard conditions at 22–25 °C and a 12 h light/12 h dark cycle. It housed six animals per cage with food and water provided adaptively for one week and high-fat diet for 4 weeks in the laboratory prior to experiment. The T2DM rats model was created by streptozocin treatment, as previously reported [15]. Briefly, the SD rats were fasted overnight before a single intraperitoneal injection of 40 mg/kg streptozocin, which was dissolved in 0.1 mol/L citric acid buffer (pH 4.2–4.5). On the third and seventh days after streptozocin injection, we took tail vein blood to determine fasting and post-meal blood glucose by Onetouch UltraEasy (Johnson, China). T2DM rats were those whom fasting blood glucose above 7.8 mmol/L and postprandial blood glucose above 16.7 mmol/L and chosen for the following studies.

### Euthanasia

According to AVMA Guidelines on Euthanasia, inhaled anesthetics are acceptable with conditions for euthanasia of small animals (< 7 kg), euthanasia was performed after exposure to the inhaled anesthetic sevoflurane at high concentrations resulting in rapid loss of consciousness.

### Key instrumentation

UPLC–MS/MS was carried out on Waters Acquity UPLC I Class (Waters, USA), which comprised a quaternary pump, an autosampler, a column oven, and an AB QTRAP 4500 mass spectrometer equipped with an electrospray ion source. Analyst version 1.6.2 was used for data acquisition and analysis. Synergy H1 multi-mode microplate reader (BioTek, USA) with Gen 5 analyzing Software was used to detect DPP-4 inhibition rate, insulin, and blood glucose.

### UPLC-MS/MS methods

#### Liquid chromatographic and mass spectrometric conditions

Chromatographic separation was achieved using an Acquity UPLC BEH C_18_ column (2.1 × 50 mm, 1.7 μm, Waters, Wexford, Ireland). The mobile phase contained 40% acetonitrile and 60% 10 mM ammonium formate/formic acid (pH 2.5), the isocratic elution was lasting 1.2 min. The flow rate was 0.35 mL/min. the column temperature was 45 °C, the auto-sampler temperature was 4 °C, and the injection volume was 10 µL. The mass spectrometer was operated in positive electrospray ionization (ESI^+^) mode. Multiple-reaction monitoring (MRM) mode monitored SAX, 5-OH SAX and Vildagliptin (VIL), for which the precursors to production ion transitions were SAX, 316.2-180.2; 5-OH SAX, 332.3-196.3; IS, 304.2-153.9. The De-clustering Potential (DP) and Collision Energy (CE) for each component were listed in Table 1.

**Table 1:** MRM of the analytes

Analytes	Q1 mass	Q3 mass	DP	CE
SAX	316.2	180.2	80.0	30.0
5-OH SAX	332.3	196.3	90.0	30.0
VIL	304.2	153.9	77.0	22.0

#### Calibrations and quality control samples

Standard stock solution of SAX, 5-OH SAX and VIL at a concentration of 1.0 mg/mL was separately prepared in 80% acetonitrile. The calibration standards samples were mixed by a series of dilution with blank rat plasma, in which the concentrations of SAX were 5, 25, 50, 100, 250, 500, 1000, 2000, 2500 ng/mL and the concentrations of 5-OH SAX were 1, 5, 10, 20, 50, 100, 200, 400, 500 ng/mL. All samples were prepared on the trial day. Internal working solution of VIL was prepared by diluting with 80% acetonitrile at a concentration of 150 ng/mL. The quality control samples (QC) were prepared as the same procedures. QC samples were made at 5/1 ng/mL (lower limit of quantitation, LLOQ), 15/3 ng/mL (low quality control, LQC), 1000/200 ng/mL (medium quality control, MQC), 2000/400 ng/mL (high quality control, HQC), 2500/500 ng/mL (low dilution quality control, LDQC) and 3500/3500 ng/mL (high dilution quality control, HDQC) for SAX/5-OH SAX respectively. All solutions were stored at − 20 °C.

#### Extraction procedure

Take 50 µL rat plasma sample into a 1.5 mL centrifuge tube, add 10 µL internal standard (IS) working solution (150 ng/mL) and 250 µL acetonitrile respectively, vortex mixed for 3 min. Then the samples were centrifuged at 13,500 rpm for 10 min at 4 °C, transfer the supernatant to a 1.5 ml centrifuge tube, which blowed dry with nitrogen at room temperature. The residue was reconstituted with 500 µL of mobile phase, vortexed-mixed for 3 min, centrifuged at 13,500 rpm for 5 min, and 10 µL of the supernatant was taken for injection.

#### Method validation procedures

The method was validated for its selectivity, linearity, accuracy, precision, recovery, matrix effect, dilution integrity, stability according to ICH Harmonised Guideline bioanalytical method validation M10 [16].

#### Selectivity

Selectivity was investigated by analyzing the chromatograms of six batches of rat blank plasma and the corresponding spiked plasma samples at LLOQ level, and plasma obtained after administration. The assay was considered to exclude the potential interference of endogenous substances at the retention times of SAX, 5-OH SAX and IS if the response was < 20% of that of the LLOQ for SAX, 5-OH SAX and < 5% of that of the IS.

#### Linearity, precision and accuracy

The calibration curves plotted the analyte concentrations against the internal standard peak area ratios for linear regression analysis. A weight of 1/x^2^, 1/x was applied to minimize the relative error for the SAX, 5-OH SAX curve fitting respectively. The accuracies of the back-calculated concentrations of each calibration standard were within 20% of the nominal concentration at the LLOQ and within 15% at all other levels. The intra-day precision and accuracy were determined using five replicates for LLOQ measurement and three concentrations of QC samples on the same day. The inter-day precision and accuracy was evaluated through five consecutive days for the same QC samples. The accuracy was expressed as a percentage of the nominal concentration, and the precision was expressed by the % of relative standard deviation. The acceptable criteria for accuracy and precision were 20% for LLOQ and the 15% for the other control samples, containing LQC, MQC and HQC.

#### Extraction recovery and matrix effects

The extraction recovery was determined by comparing the peak area of obtained from the extracted spiked sample with that of the post-extracted spiked sample at LQC, MQC, HQC and IS (five replicates for each concentration). The matrix effect factors (6 sources, 3 replicates) was evaluated by comparing the peak area of the post-extracted spiked sample with those of samples spiked with water at LQC and HQC. Calculate the matrix factors of SAX, 5-OH SAX, and IS, divide the matrix factors of SAX, 5-OH SAX by the matrix factor of IS, and obtain the IS-normalized matrix factor. The assay precision for the recovery and matrix effects should be within 15% of the relative standard deviation.

#### Stability

The stability of analytes in QC plasma was evaluated by analyzing replicates (*n* = 3) of LQC, MQC, HQC samples placed on storage for 4 h at room temperature (25 °C), for 12 h at 4 °C, for 7 days at −80 °C, and after three freeze-thaw cycles from −80 °C to room temperature. The auto-sampler stability was studied by reanalyzing the extracted samples kept in the auto-sampler at 4 °C for 16 h. Samples were considered stable if assay values were within ±15% of the nominal values.

#### Dilution integrity

Using the same source of blank rat plasma, the dilution integrity was evaluated by diluting LDQC and HDQC samples. The dilution factor of SAX was 2, i.e., the dilution sample obtained by doubling dilution LDQC and HDQC samples once with blank plasma, and the dilution factor of 5-OH SAX was 8, i.e., the dilution sample obtained by doubling dilution LDQC and HDQC samples three times with blank plasma. The concentrations of SAX and 5-OH SAX in the diluted samples were analyzed, and the above operations were repeated five times to evaluate the dilution integrity of the method. The average accuracy of the dilution control should be within ± 15% of the labeled value, and the precision should not exceed 15%.

#### Pharmacokinetic measurements

T2DM rats were randomly separated into SAX control group (*n* = 3, i.g. 10 mg/kg 0.5% CMC-Na), SAX group (*n* = 3, i.g. 10 mg/kg SAX dissolved in 0.5% CMC-Na), 5-OH SAX control group (*n* = 3, i.v. 0.5 mg/kg saline) and 5-OH SAX group (*n* = 3, i.v. 0.5 mg/kg 5-OH SAX dissolved in saline). Blood samples were collected at 0, 0.03 (only for 5-OH SAX), 0.08, 0.17, 0.25, 0.5, 0.75, 1, 1.5, 2, 3, 4, 6, 8, 10, 12 and 24 h after dosing. Plasma was obtained by centrifugation at 3000 r/min and stored at −80 °C until analysis.

Plasma concentrations of analytes were determined using the UPLC-MS/MS method above. The plasma standard curves ranged from 5 to 2500 ng/mL for SAX and from 1 to 500 ng/mL for 5-OH SAX and the specificity, linearity, precision, accuracy, recovery, and stability of the method were eligible.

#### The measurement methods of insulin concentration and glucose concentration

Insulin concentration in the plasma was determined by Rat INS ELISA Kit (Zeye Biotechnology Co., Ltd., Shanghai, China). All the procedures were conducted according to manufacturers’ instructions. Test specimens (plasma samples, blank and calibration samples) were added to an antibody-coated 96-well plate followed by addition of biological agent and enzyme conjugate solution, these mixtures incubated 30 min at 37 °C. The plate was then washed after incubation and a mixture of chromogenic reagent A and B was added. Optical density values at 450 nm were measured after terminating the reaction, and insulin concentrations were calculated according to the standard curve within a range of 0–20 mU/L (0, 1.25, 2.5, 5, 10, 20 mU/L).

Glucose concentration in the plasma was determined by Glucose test kit (glucose oxidase method, Rongsheng biological Pharmaceutical Co.,Ltd, Shanghai, China). All the procedures were conducted according to manufacturers’ instructions. All test specimens (plasma samples, blank and calibration samples) were done in flat bottom 96 well microplates; 2.5 µL serum or calibration, 250 µL solution were pipetted into each well of the microplate, and the blank control contain water. The results were tested at 37 °C for 10 min and an absorbance value of 505 nm in a continuous monitoring micro plate reader. Glucose concentration (mmol/L) = (sample tube absorbent/calibration tube absorbent) × calibration fluid concentration (5.55 mmol/L). All experiments were repeated two duplicates.

#### The DPP-4 inhibitory measurement methods

Any polypeptide with proline (Pro) or alanine (Ala) at the second position of the N-terminal of the structure is the main substrate for DPP-4 [17]. DPP-4 activity was examined using Gly-Pro-pNA as the substrate for DPP-4 in this study. Briefly, DPP-4 can cleavage Gly-Pro-pNA to produce Gly-Pro and *p*-nitroanilide, which was absorbed at 405 nm. It was proportional to the amount of DPP-4 activity in the plasma. DPP-4 activity levels were expressed as the amount of cleaved pNA per minute per ml. In order to explore the optimal reaction conditions, we carried out the following optimizations.

#### Effects of pH, temperature, time and Gly-Pro-pNA concentration on DDP-4 activity

The effects of external factors on the DPP-4 activity were tested under the following conditions. To examine the effect of Gly-Pro-pNA concentration, DDP-4 activity was tested at 0.06, 0.12, 0.24, 0.34, 0.68, 0.95 and 1.90 mM at 37 °C, pH 8.0. Optical density (OD) values were measured every 5 min for 4 h. To determine the effect of pH and reaction temperature, different pH values (7.0, 7.4, 8.0, 8.5, 8.7 and 9.0) were used for the assay under different reaction temperature at 25 °C, 30 °C, 35 °C, 37 °C, and 40 °C at 1.90 mM Gly-Pro-pNA. All experiments were repeated two duplicates.

#### Linearity, LLOQ, accuracy and precision of plasma pNA

The six-point linear calibration curve was constructed by plotting the OD values vs. the theoretical plasma pNA concentrations over the range of 0.0625–1 mg/mL. The lower limit of quantitation (LLOQ) was defined as the concentration where the accuracy and precision were up to ±20% Standard Deviation (SD, %) and coefficient of variation (CV, %), respectively. Accuracy and intra- and inter-day precision were assessed by analyzing five consecutive batches containing calibration curve standards and two replicates of each QC level (low, medium and high). The accuracy was expressed as SD (%), and the precision as CV (%). Both SD and CV were expected to be within ±15% to be acceptable.

#### Pharmacodynamic measurements

According to the above optimized conditions, the final reaction conditions were determined as follows: the concentration of Gly-Pro-pNA was 1.9 mM, reaction conditions were 40 °C and pH 8.5, kinetic monitoring for 2.5 h, dynamic determination of absorbance standard curve every 5 min. Standard curve was measured as the following conditions: 50 µL gradient concentration pNA (0.0625, 0.125, 0.25, 0.5, 0.75, 1 mg/mL), 40 µL assay buffer (50 mM Tris-HCl, pH 8.5) and 10 µL blank serum were pipetted into each well of the flat bottom 96 well microplates, then determined the absorbance values after incubating at 40 °C for 10 min. Blank hole were measured in this condition: 10 µL blank plasma sample, 40 µL assay buffer and 50 µL assay buffer. Sample holes were obtained in 10 µL plasma samples (each time point), 40 µL assay buffer and 50 µL 1.9 mM Gly-Pro-pNA (dissolved in 1 mM EDTA and 50 mM Tris-HCl, pH 8.7). All holes were pipetted. Kinetic monitoring A_0_ (blank hole) and A_i_ (sample holes). According to linearity, obtained C_0_ and C_i_, calculating ΔC. Enzyme activity unit was equal to ΔC/5 (mL/L/min pNA production). DPP-4 inhibitory rate = 1-U_i_/U_0_.

#### PK model

**Fig. 1:**
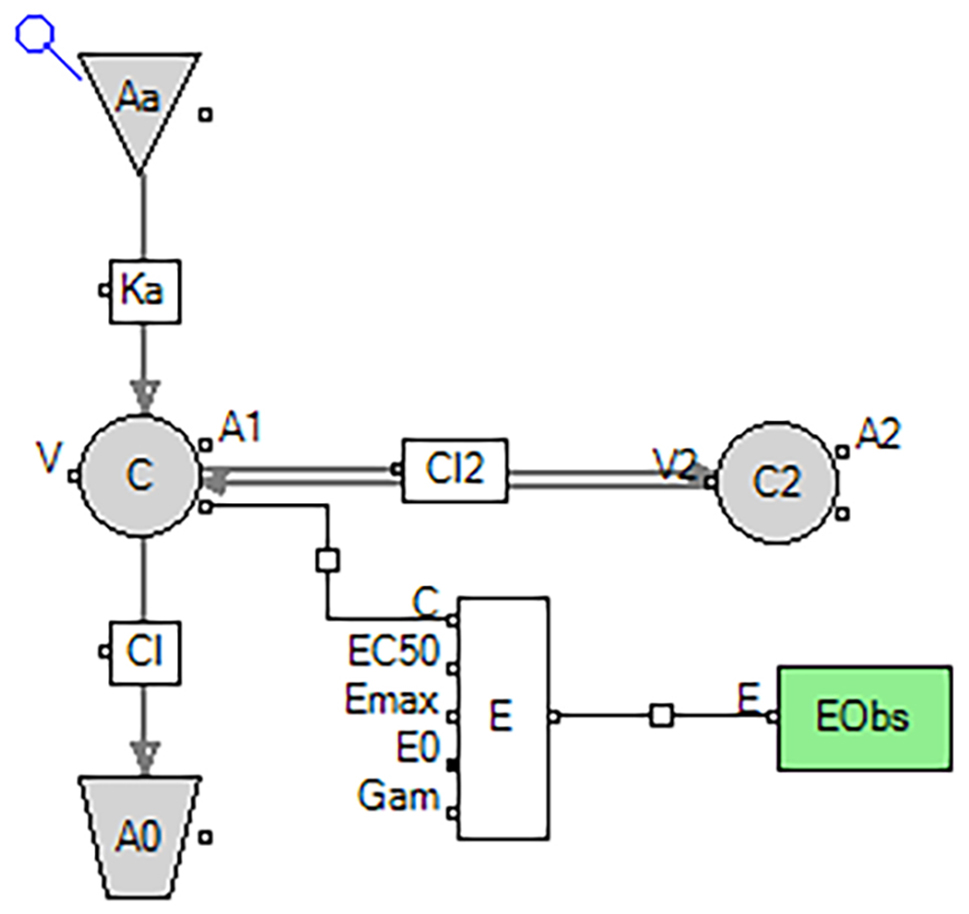
Schematic representation of the PK/PD model of SAX in T2DM rats

**Fig. 2:**
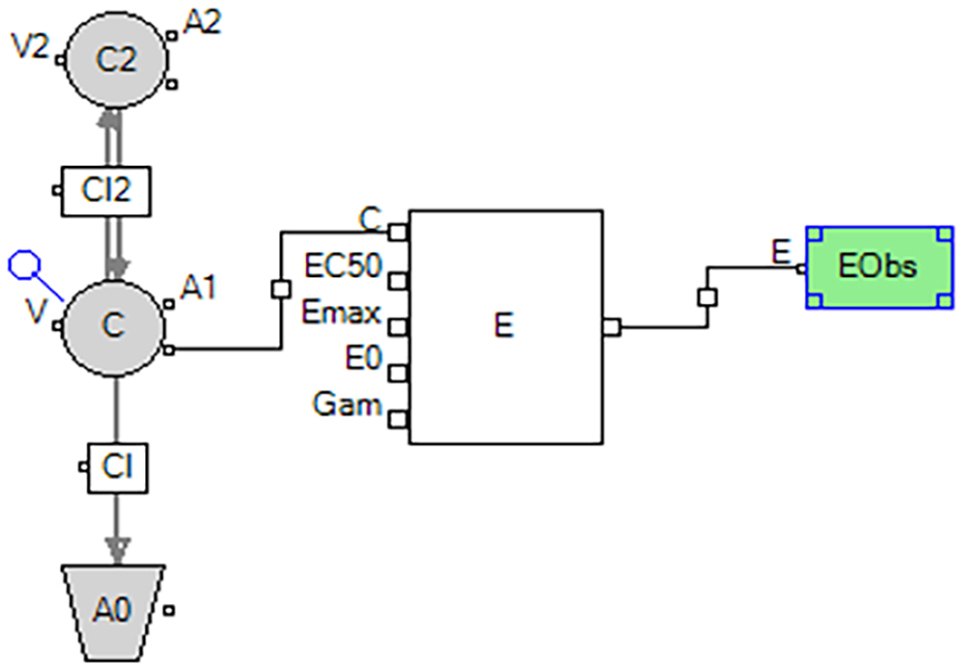
Schematic representation of the PK/PD model of 5-OH SAX in T2DM rats

A extravascular two-compartment model with first order absorption with additive residual error (Fig. 1) and a intravenous injection two-compartment model with additive residual error (Fig. 2) was used to characterize the PK of SAX and 5-OH SAX in T2DM rats, respectively.

1. The differential equations of the SAX PK model are as follows:

$$\frac{{d{A_1}}}{{dt}}=F \cdot {K_a} \cdot {A_a} \cdot +\frac{{C{L_2}}}{{{V_2}}} \cdot {A_2} - \frac{{C{L_2}}}{V} \cdot {A_1} - \frac{{CL}}{V} \cdot {A_1}$$

$$\frac{{d{A_2}}}{{dt}}=\frac{{C{L_2}}}{V} \cdot {A_1} - \frac{{C{L_2}}}{{{V_2}}} \cdot {A_2}$$

$$K=\frac{{CL}}{V}$$

Where A_a_, A_1_ and A_2_ represent the amounts of SAX in the absorption, central and peripheral compartments, respectively; Ka represents the first order absorption rate; CL is the systemic clearance; CL_2_ is the clearance rate from central compartment to peripheral compartment; V represents the apparent volume of distribution; V_2_ represents the apparent distribution volume of peripheral compartment and C is the concentration of SAX in the central compartment.

(2). The differential equations of the 5-OH SAX PK model are as follows:

$$\frac{{d{A_1}}}{{dt}}=\frac{{C{L_2}}}{{{V_2}}} \cdot {A_2} - \frac{{C{L_2}}}{V} \cdot {A_1} - \frac{{CL}}{V} \cdot {A_1}$$

$$\frac{{d{A_1}}}{{dt}}=\frac{{C{L_2}}}{V} \cdot {A_1} - \frac{{C{L_2}}}{{{V_2}}} \cdot {A_2}$$

$$K=\frac{{CL}}{V}$$

Where A_1_ and A_2_ represent the amounts of 5-OH SAX in the central and peripheral compartments, respectively; CL is the systemic clearance; CL_2_ is the clearance rate from central compartment to peripheral compartment; V represents the apparent volume of distribution; V_2_ represents the apparent distribution volume of peripheral compartment and C is the concentration of 5-OH SAX in the central compartment.

#### PK/PD link model

PK/PD models of were established to quantitatively describe the effects of SAX and 5-OH SAX on DPP-4 activity in T2DM rats, respectively. The diagrams of the PK/PD model are illustrated in Figs. 1 and 2. The Hill’s function accompanied by directly linked model was employed to describe the inhibitory effect of 5-OH SAX on DPP-4 activity:

$$E={E_0}+{E_{{\text{max}}}} \cdot \frac{{{C^\gamma }_{{}}}}{{E{C_{50}}^{\gamma }+{C^\gamma }}}$$

Where E represents the inhibition ratio of DPP-4 activity after drug treatment; E_0_ means that the basic effect without administration; E_max_ and EC_50_ represent the maximum DPP-4 inhibition ratio and the concentration of 5-OH SAX to exhibit half the maximum inhibitory effect; C is the plasma concentration of 5-OH SAX, and Gam(*γ*) is a shape factor.

Another function means the inhibitory effect of parent SAX on DPP-4 activity, the letters get the same meaning as above, the difference is that there is no basic effect. Its function is as follow:

$$E={E_{{\text{max}}}} \cdot \frac{{{C_{}}^{\gamma }}}{{E{C_{50}}^{\gamma }+{C_{}}^{\gamma }}}$$

#### Statistical analysis

Modeling construction and PK/PD parameters calculation were performed using WinNolin 8.1 (Phoenix Certara, Louis, USA). The insulin, glucose and DPP-4 inhibitory rate were statistically analyzed by SPSS 21.0 software, and the comparison between the control group and trial group was performed by *t*-test or non-parametric test. The hypothesis test level was determined by *α* = 0.05, and *P* < 0.05 was statistically significant.

## Results

### Verification of UPLC-MS/MS method

#### Selectivity and specificity

**Fig. 3:**
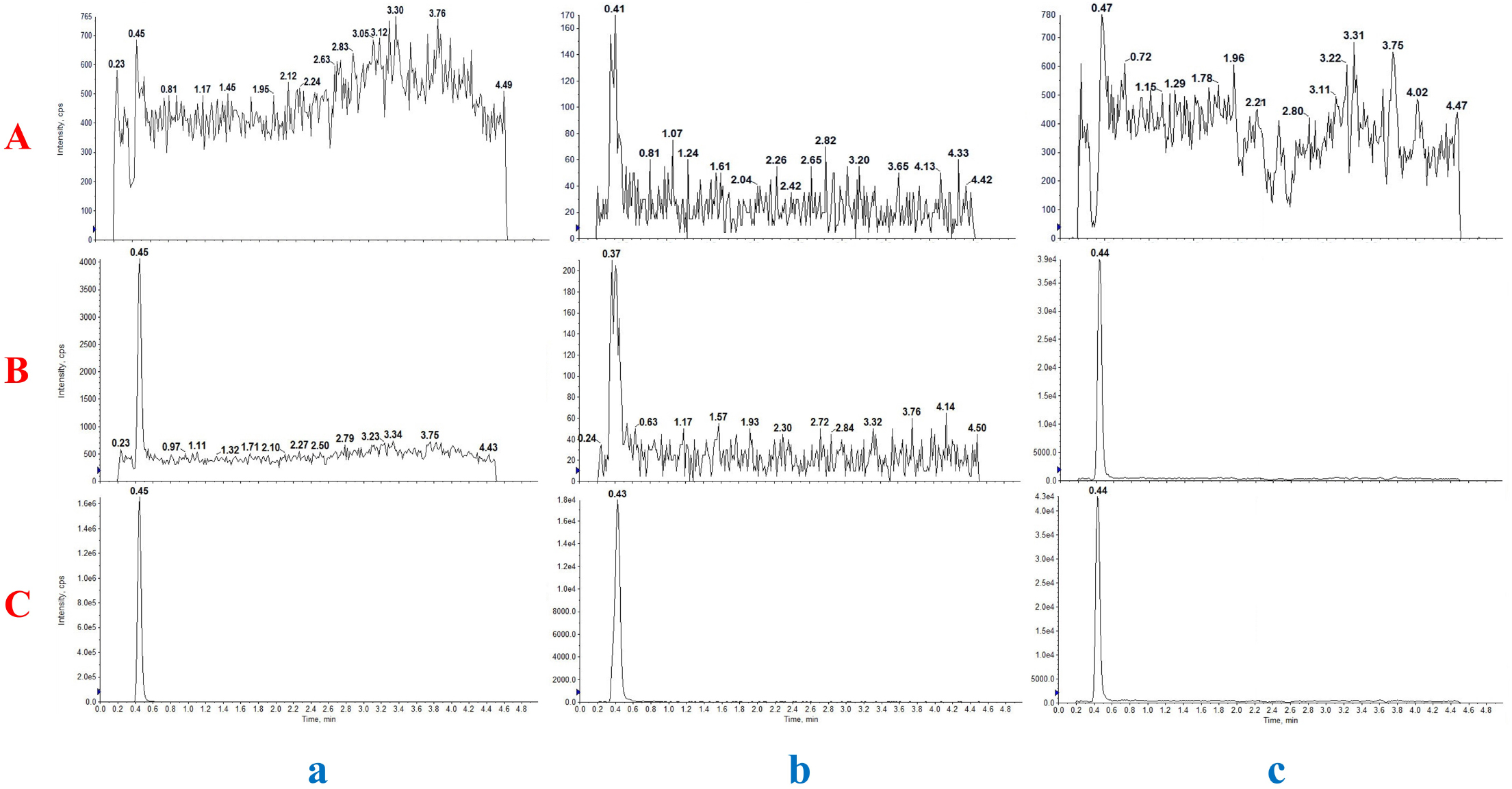
Chromatograms of blank rat plasma (**A**), blank rat plasma spiked with SAX, 5-OH SAX at LLOQ (5/1 ng/mL, respectively) (**B**), and plasma sample from a rat at 1.5 h after oral administration of 10 mg/kg SAX (**C**). Channel a, SAX; b, 5-OH SAX; c, IS

Retention times of SAX, 5-OH SAX and IS were 0.45, 0.43 and 0.44 min, respectively. No significant interference peaks were observed in the retention regions of both compounds. Figure 3 shows typical chromatograms from blank plasma, plasma spiked with SAX and 5-OH SAX at the LLOQ, and plasma obtained 1.5 h after administration.

#### Linearity, accuracy and precision

Data for linear interval, calibration equation and Correlation coefficients (*R*^2^) of the method for SAX and 5-OH SAX determination are summarized in Table 2. The *R*^2^ of calibration curves in all inter-run cases were >0.999 over the concentration range from 5 to 2500 ng/mL for SAX and 1-500 ng/ml for 5-OH SAX. The LLOQ of SAX and 5-OH SAX was 5 and 1 ng/mL in this assay. Table 3 presents the results for accuracy and precision evaluation. For SAX, intra- and inter-day accuracy ranges from 99.45 to 101.24% and 99.28–99.99%, intra- and inter-day precisions were 1.25–5.63 and 1.97–6.21% (RSD), respectively. For 5-OH SAX, intra- and inter-day accuracy ranges from 100.46 to 106.20% and 100.05–105.47%, intra- and inter-day precisions were 1.10–7.43 and 3.71–9.11% (RSD), respectively. These results indicate that it is a precise and accurate method.

**Table 2:** Linear range, equation, and correlation coefficient of SAX and 5-OH SAX

Analyte	Linear (*n* = 5)			LLOQ (ng/mL)	Weighting scheme
	Linear interval (ng/mL)	*R* ^2^	Calibration equation		
SAX	5-2500	0.9996	y = 0.0216x − 0.0084	5	1/x^2^
5-OH SAX	1-500	0.9998	y = 0.0017x + 0.0058	1	1/x

**Table 3:** Intra-day and inter-day precision and accuracy of SAX and 5-OH SAX

Analytes	Norminal concentration (ng/mL)	Intra-day (*n* = 5)			Inter-day (*n* = 5)		
		Measured concentration (ng/mL)	Accuracy (%)	Precision (RSD, %)	Measured concentration (ng/mL)	Accuracy (%)	Precision (RSD, %)
SAX	5	5.06	101.24	5.63	4.97	99.32	6.21
	15	14.92	99.45	2.79	14.93	99.55	3.40
	1000	1004.60	100.46	1.25	999.88	99.99	1.97
	2000	2003.18	100.16	2.21	1985.63	99.28	3.01
5-OH SAX	1	1.06	106.20	7.43	1.03	103.00	9.11
	3	3.01	100.47	3.88	3.16	105.47	6.71
	200	200.93	100.46	1.82	203.78	101.89	3.71
	400	402.18	100.55	1.10	400.21	100.05	4.21

#### Extraction recovery and matrix effect

Table 4 presents the results for extraction recovery evaluation. The overall recovery efficiency was 82.58–93.37% for SAX, 89.27–96.18% for 5-OH SAX and 92.19% for IS. The recovery result indicates that acetonitrile is a feasible and appropriate medium for SAX, 5-OH SAX and VIL extraction. Table 5 shows that the average matrix effects of LQC and HQC SAX for six batches were 93.26% and 84.24%, respectively. The IS-normalized matrix effects were 109.39% and 100.42% for LQC and HQC, respectively. The average matrix effects of LQC and HQC 5-OH SAX for six batches were 86.61% and 85.01%, respectively, with IS-normalized matrix effects of 104% and 102.12%, respectively. The precision of each QC sample was less than 15%. This result indicates that matrix components did not significantly alter the performance of chromatography or the ionization of analytes; the matrix effect on the ionization of analytes was not serious under these experimental conditions and could be neglected.

**Table 4:** Extraction recovery evaluation of SAX and 5-OH SAX

Analytes	Norminal concentration (ng/mL)	Extraction recovery (*n* = 5) (%)		
		Mean	SD	RSD
SAX	15	82.58	3.41	4.13
	1000	93.37	2.07	2.22
	2000	92.96	2.31	2.48
5-OH SAX	3	96.18	3.18	3.30
	200	91.86	2.80	3.05
	400	89.27	3.90	4.37
VIL	150	92.19	1.91	2.07

**Table 5:** Matrix effect evaluation of SAX and 5-OH SAX (*n* = 3, sources = 6)

Analytes	Norminal concentration (ng/mL)	Analyte matrix effect (%)			IS normalization analyte matrix effect (%)		
		Mean	SD	RSD	Mean	SD	RSD
SAX	15	92.36	2.96	3.21	109.39	5.56	5.08
	2000	84.26	1.50	1.78	100.42	4.33	4.31
5-OH SAX	3	86.61	3.32	3.83	104.00	5.34	5.13
	400	85.01	3.60	4.23	102.12	4.13	4.04

#### Stability

SAX and 5-OH SAX remained stable in different conditions including room temperature (25 °C) for 4 h, 4 °C for 12 h, −80 °C for 7 days, three freeze-thaw cycles, and autosampler at 4 °C for up to 16 h. The results are shown in Table 6.

**Table 6:** Stability evaluation of SAX and 5-OH SAX (*n* = 3)

Analytes	Spiked concentration (ng/mL)	Freshly preparations (%)	Room temperature for 4 h (%)	4 °C for 12 h (%)	−80 °C for 7 days (%)	Freeze-thaw three cycles (%)	Autosampler at 4 °C for up to 16 h (%)
SAX	15	100.62 ± 3.08	99.02 ± 2.02	100.60 ± 2.29	98.56 ± 2.14	96.71 ± 1.93	99.02 ± 2.00
	2000	98.99 ± 2.10	99.59 ± 3.47	99.09 ± 2.02	98.07 ± 2.07	95.84 ± 1.52	98.05 ± 2.04
5-OH SAX	3	100.67 ± 4.16	99.78 ± 2.22	100.67 ± 2.52	95.11 ± 4.55	94.89 ± 2.91	100.44 ± 3.10
	400	101.04 ± 2.71	98.51 ± 2.19	99.32 ± 2.66	97.49 ± 2.52	95.79 ± 2.16	101.22 ± 2.47

#### Dilution integrity

Table 7 shows that for LDQC and HDQC samples, dilution with dilution factors for SAX and 5-OH SAX resulted in average back-calculated values around 100%, with precision less than 15%. This indicates that dilution of samples according to the corresponding dilution factors does not affect the accuracy and precision of the method.

**Table 7:** Dilution Integrity analyses of SAX and 5-OH SAX (*n* = 5)

Analytes	Norminal concentration (ng/mL)	Dilution factor	Measured concentration (ng/mL)	Accuracy (%)	Precision (RSD, %)
SAX	2500	2	2500.08	100.00	2.34
	3500	2	3500.28	100.01	2.45
5-OH SAX	500	8	503.09	100.62	3.78
	3500	8	3529.75	100.85	4.06

### Partial verification of optical analysis method of DPP-4

#### Optimal reaction time, substrate reaction concentration and pH

**Fig. 4:**
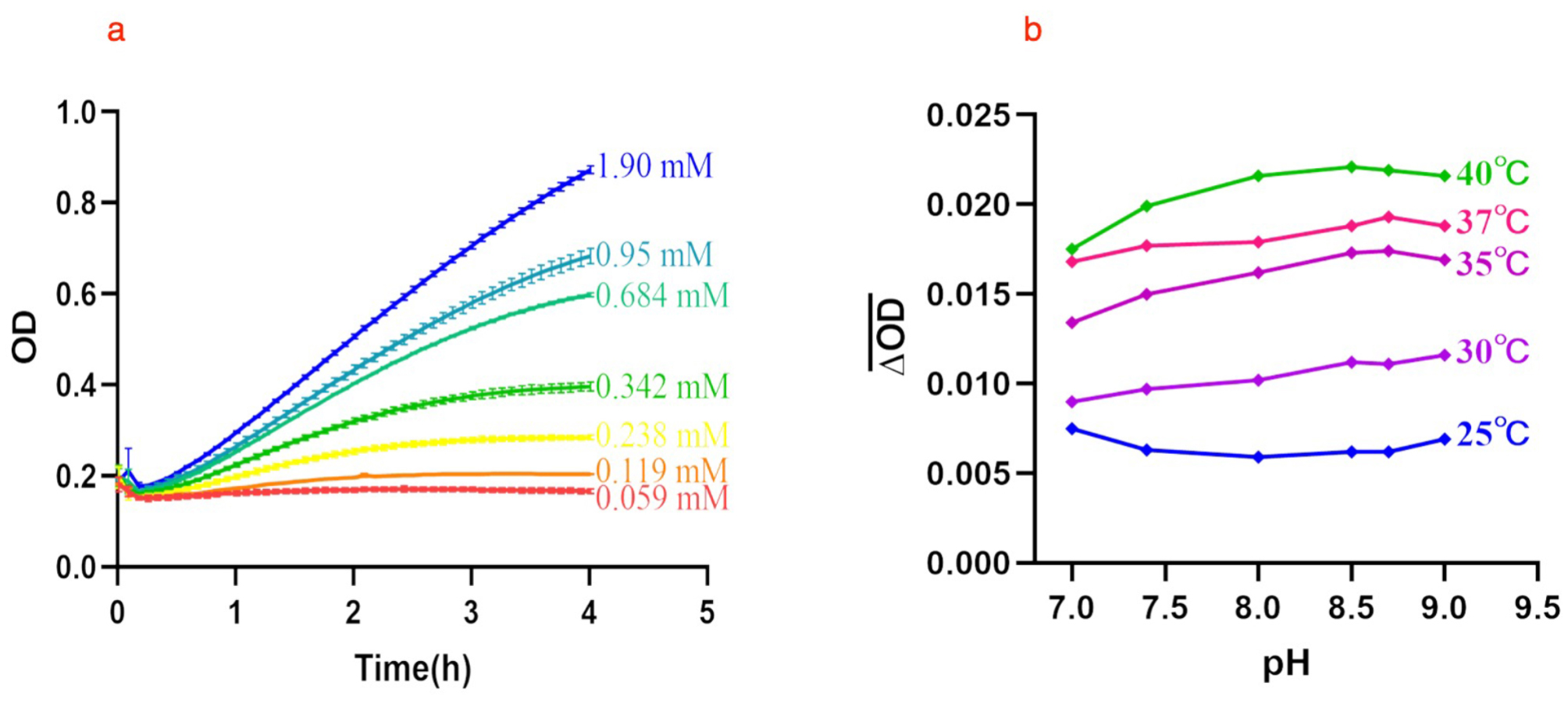
(**a**) The time-OD value relationship curves at each concentration. (**b**) The pH-$\overline {{\Delta OD}}$ value relationship curves at different temperatures

Under the reaction system of 37 °C and pH 8.0, the OD values of pNA were measured dynamically every 5 min for 4 h. Taking the reaction time as the abscissa and the OD values as the ordinate, draw the time-OD values relationship curve at each concentration (Fig. 4a). When substrate concentrations were below 0.342 mM, the OD values were small and increased slowly as the reaction time increases. While the concentrations greater than or equal to 0.684 mM, the OD values change sped up. When substrate concentration was 1.9 mM, the increase was the most obvious. And according to the optical analysis, the OD values is in the range of 0.2–0.8, the accuracy is the best. So, in order to ensure the smooth progress of the reaction, we chose 1.9 mM as the final reaction substrate concentration. At the same time, taking 1.9 mM Gly-Pro-pNA as an example, it was found that the OD values increased significantly at 1.5–3 h, so the linear best segment (1.45–2.05 h) was selected for data analysis and 2.5 h as the final reaction time.

Explore the impact of different pH buffer systems on the hydrolysis reaction at different temperatures, taking the pH as the abscissa and the average change in absorbance every 5 min as the ordinate, draw the pH-$\overline {{\Delta OD}}$value relationship curves at different temperatures. As shown in the Fig. 4b, the $\overline {{\Delta OD}}$ increases with the increase of temperature and the change is most obvious at 40 °C. On the side, pH values have little effect on the reaction, the $\overline {{\Delta OD}}$ was the largest when pH 8.5 at 40 °C. So, we selected 40 °C and pH 8.5 as the reaction temperature and buffer system.

#### Linearity, LLOQ, accuracy and precision of plasma pNA

Correlation coefficients (*R*^2^) of plasma pNA calibration curves (*Y* = 1.107*X* + 0.0067) in all inter-run cases were > 0.99 over the concentration range from 0.0625 to 1.0 mg/mL. The LLOQ of pNA was 0.0625 mg/mL in this assay. The acceptable criteria for accuracy and precision were 20% for LLOQ and the 15% for the other control samples, low (LQC), medium (MQC), and high (HQC) concentrations. Table 8 presents the results for accuracy and precision evaluation. Accuracy and precisions were 96.53–98.62 and 4.58–9.92% for intra-day, 94.11–98.86 and 4.00–8.58% for inter-day. These results indicate that it is a precise and accurate method.

**Table 8:** Intra-day and inter-day precision and accuracy of the plasma pNA

Nominal concentration (mg/mL)	Intra-day			Inter-day		
	Measured concentration (mg/mL)	Accuracy (%)	RSD (%)	Measured concentration (mg/mL)	Accuracy (%)	RSD (%)
0.0625	0.0603	96.53	9.92	0.0588	94.11	8.58
0.125	0.1232	98.62	4.58	0.1234	98.73	4.00
0.25	0.2460	98.38	8.43	0.2472	98.86	5.66
0.75	0.7256	96.74	9.45	0.7296	97.28	1.42

#### PK/PD link model

According to the technical guidelines for non-clinical pharmacokinetic research of chemical drugs, the entire sampling time should last at least until the blood concentration is 1/10 − 1/20 of *C*_max_. Therefore, for the intravenous 5-OH SAX group, the first 2 h was selected for model establishment and gavage SAX group, the first 4 h sampling points were selected for model fitting.

#### PK model of 5-OH SAX in T2DM rats

**Fig. 5:**
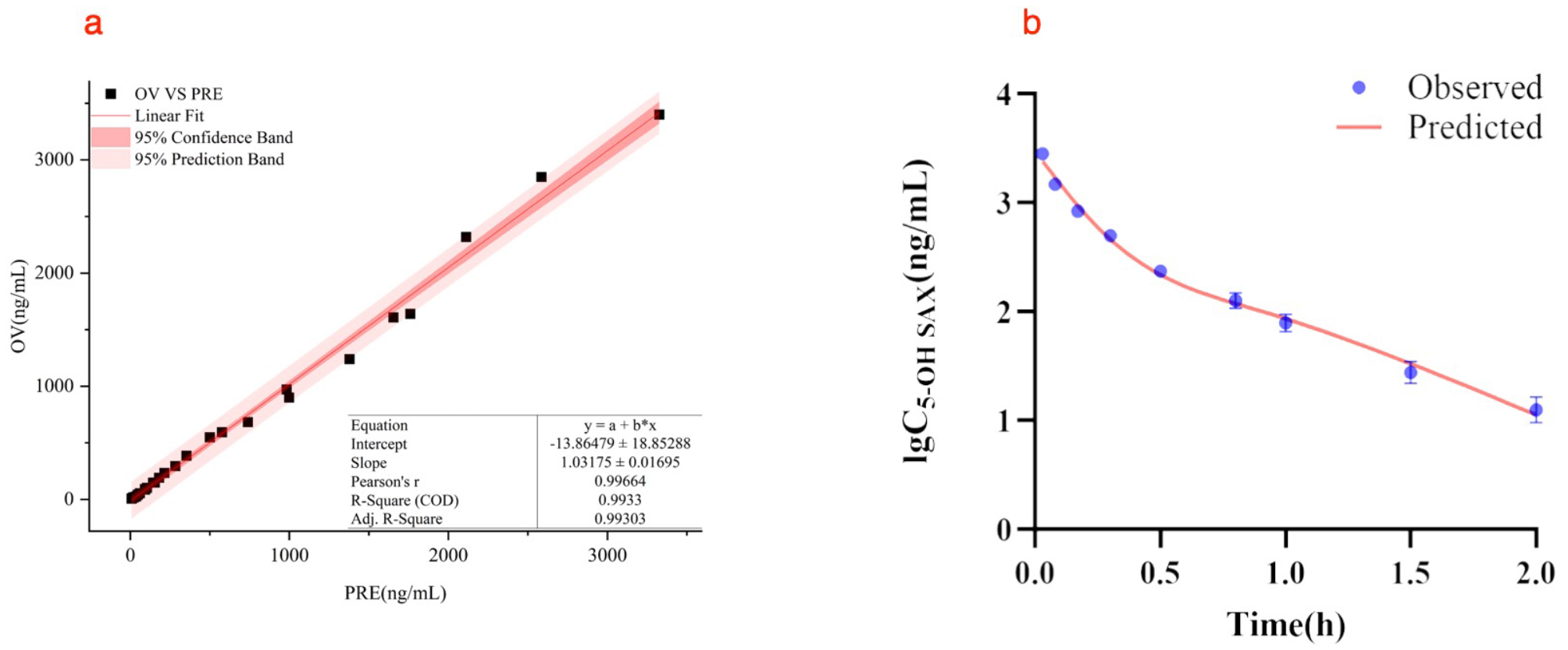
(**a**) Observed 5-OH SAX plasma concentrations versus predicted data. The linear line is red and the OV vs. PRE points are black solid square. (**b**) The PK profiles of 5-OH SAX in T2DM rats following a tail vein dose of 0.5 mg/kg. The blue solid points represent observed concentration data (*n* = 3). The red solid line represents model fitting curve

In the linear fitting curve plot, the distributions of the observed values (OV) versus predicted values (PRE) showed symmetry besides the linear line (Fig. 5a). The pearson’s *r* was equal to 0.9966, which meant the observed values and predicted values were obviously positively correlated. Most of the observed values fell in the 95% confidence band (CB) of the predictions, implying that the final model adequately described and predicted the PK profiles.

The observed and model-fitted lg plasma concentration versus time course of 5-OH SAX in T2DM rats at a tail vein dose of 0.5 mg/kg is shown in Fig. 5b. The PK profile was characterized well by an intravenous injection two-compartment model. The estimated PK parameters are summarized in Table 9. An additive residual error model was selected to account for the differences between observed and predicted concentration values.

**Table 9:** Parameters of the PK model of 5-OH SAX in T2DM rats (*n* = 3)

Parameter	Mean	SD	CV (%)
C_max_ (ng/mL)	3716.88	1061.39	28.56
AUC (ng*h/mL)	585.04	121.83	20.82
t_1/2a_ (h)	0.06	0.01	16.50
t_1/2β_ (h)	0.36	0.03	7.88
V (ml/kg)	141.49	36.91	26.09
V_2_ (ml/kg)	137.81	13.03	9.46
CL (ml/h/kg)	882.19	199.34	22.60
CL_2_ (ml/h/kg)	471.60	56.89	12.06
K_12_ (1/h)	3.51	1.16	33.05
K_21_ (1/h)	3.42	0.11	3.16

*C*_max_ maximum plasma concentration, *AUC* area under the curve, *t*_1/2α_ distribution phase half-life, *t*_1/2β_ elimination half-life, *V* apparent distribution volume of central compartment, *V*_*2*_ apparent distribution volume of peripheral compartment, *CL* the clearance rate of central compartment, *CL*_*2*_ the clearance rate of peripheral compartment, *K*_*12*_ rate constant from compartment 1 to compartment 2, *K*_*21*_ rate constant from compartment 2 to compartment 1

#### PK/PD link model of 5-OH SAX in T2DM rats

**Fig. 6:**
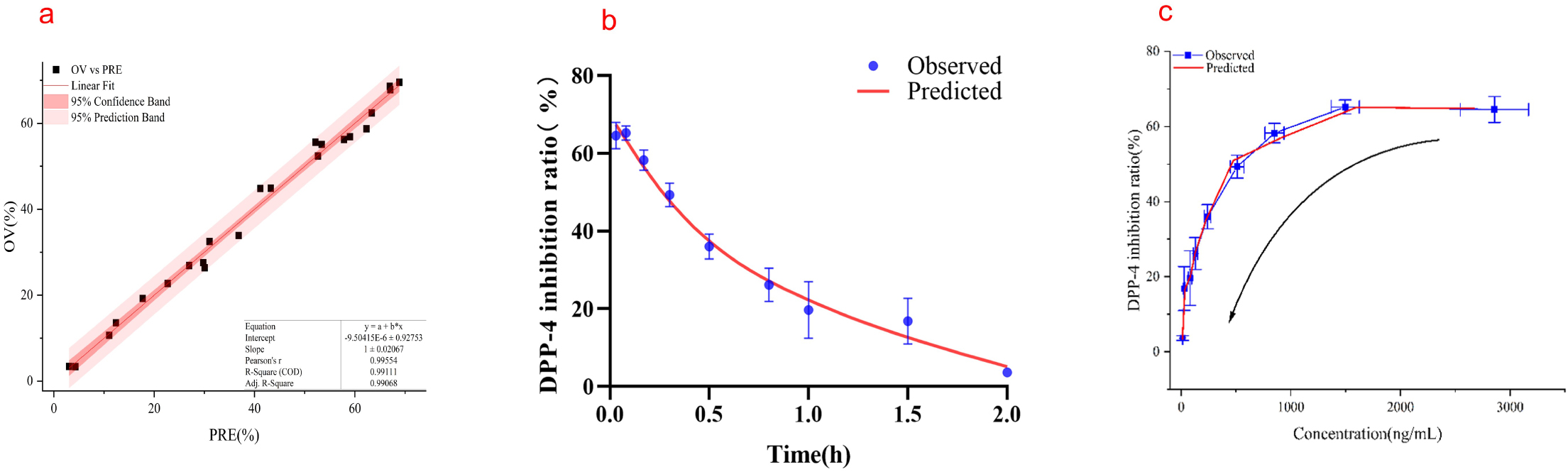
(**a**) Observed 5-OH SAX DPP-4 inhibition ratio versus predicted data. The linear line is red and the OV vs. PRE points are black solid square. (**b**) The PD profiles of 5-OH SAX in T2DM rats following a tail vein dose of 0.5 mg/kg. The blue solid points represent observed DPP-4 inhibitory (*n*=3). The red solid line represents model fitting curve. (**c**) The dose-effect curve of 5-OH SAX in T2DM rats. The arrow indicates that the efficacy of the drug shows a downward trend with the time of administration

Based on the determination of PK parameters, the sigmoidal E_max_ with E_0_ model was selected to directly fit the DPP-4 inhibition ratio model after 5-OH SAX intravenous injection. In the linear fitting curve plot (Fig. 6a), the pearson’s *r* was equal to 0.9955, meant the high goodness of fit. Most of the observed points also fell in the 95% prediction band (PB) of the predictions, implying that the final model adequately described and predicted the PK/PD profiles.

The observed and model-fitted DPP-4 inhibition ratio versus time course of 5-OH SAX in T2DM rats is shown in Fig. 6b. The concentration-effect curve is shown in the Fig. 6c, the greater the plasma drug concentration, the higher the inhibition rate, and there is no hysteresis effect after administration. The estimated PD parameters are summarized in Table 10.

**Table 10:** Parameters of the PD model of 5-OH SAX in T2DM rats (*n* = 3)

Parameter	Units	Mean	SD	CV
EC_50_	ng/mL	251.74	97.31	38.65
Gam		1.31	0.26	20.10
E_0_		7.03	12.81	182.30
E_max_		60.88	12.54	20.60
stdev0		1.97	0.15	7.79

*EC*_*50*_ median effective concentration, *Gam* slope parameter of S-curve, *E*_*max*_ maximum effect of drug, *stdev0* fitting residual error

#### PK model of parent SAX in T2DM rats

**Fig. 7:**
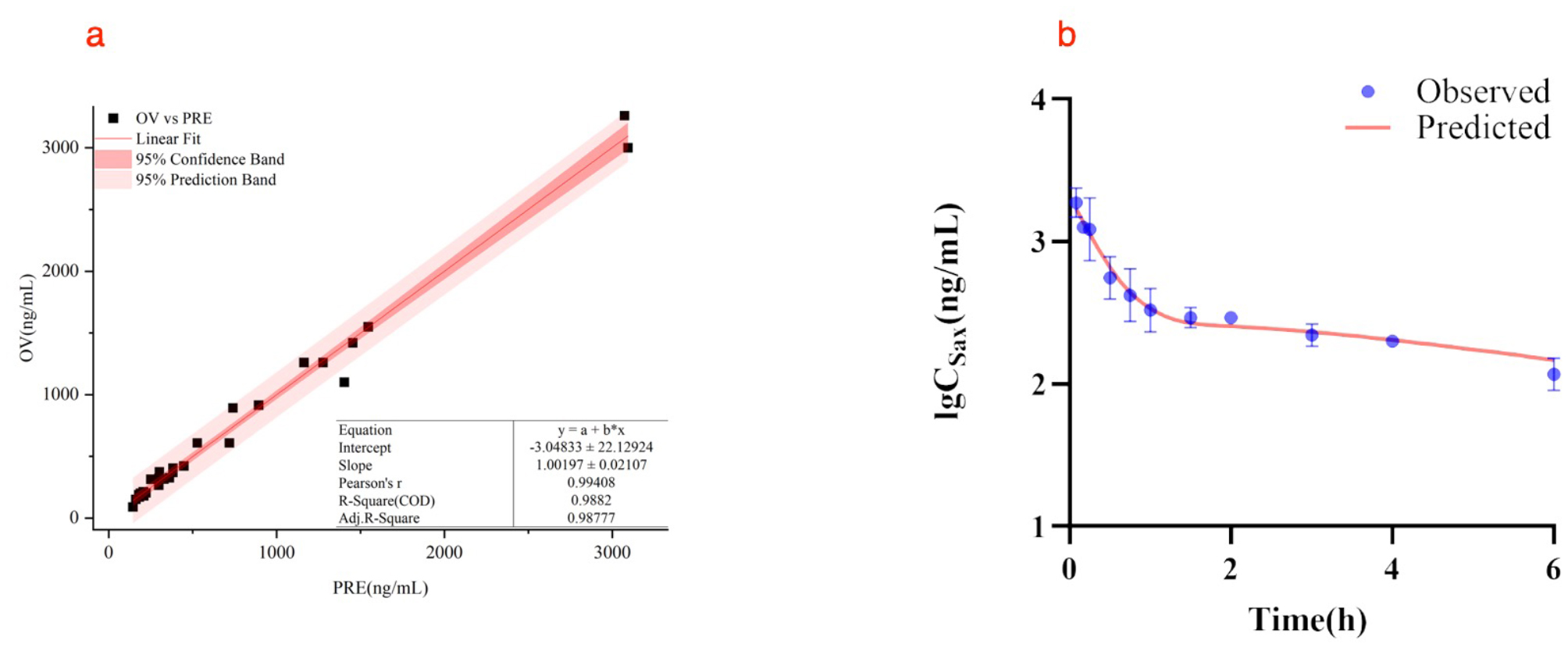
(**a**) Observed SAX plasma concentrations versus predicted data. The linear line is red and the OV vs. PRE points are black solid square. (**b**) PK profiles of SAX in T2DM rats following a intragastric dose of 10 mg/kg. The blue solid points represent observed concentration data (*n* = 3). The red solid line represents model fitting curve

In the linear fitting curve plot, the OV versus PRE points is symmetry distribution besides the linear line (Fig. 7a). The pearson’s *r* was equal to 0.9941, which meant the good relevance. The 95% CB and the 95% PB imply that the final model is appropriated.

The observed and model-fitted lg plasma concentration versus time course of SAX in T2DM rats at an intragastric dose of 10 mg/kg is shown in Fig. 7b. The PK profile was characterized well by an extravascular two-compartment model with first order absorption. The estimated PK parameters are summarized in Table 11. An additive residual error model was selected to account for the differences between observed and predicted concentration values.

**Table 11:** Parameters of the PK model of SAX in T2DM rats (*n* = 3)

Parameter	Mean	SD	CV (%)
C_max_ (ng/mL)	2197.13	1205.08	54.85
T_max_ (h)	0.11	0.03	27.63
AUC (ng*h/mL)	3282.06	1085.78	33.08
t_1/2Ka_ (h)	0.07	0.03	37.50
t_1/2a_ (h)	0.08	0.02	20.79
t_1/2β_ (h)	6.13	6.20	101.21
V (mL/kg)	2307.24	1097.52	47.57
V_2_ (mL/kg)	18393.30	16552.77	89.99
CL (mL/h/kg)	3245.80	902.06	27.79
CL_2_ (mL/h/kg)	17090.42	11724.77	68.60
K_12_ (1/h)	6.62	3.06	46.22
K_21_ (1/h)	1.07	0.40	37.47

*C*_max_ maximum plasma concentration, *T*_max_ time to maximum plasma concentration, *AUC* area under the curve, *t*_1/2Kα_ absorption phase half-life, *t*_*1/2α*_ distribution phase half-life, *t*_*1/2β*_ elimination half-life, *V* apparent distribution volume of central compartment, *V*_*2*_ apparent distribution volume of peripheral compartment, *CL* the clearance rate of central compartment, *CL*_*2*_ the clearance rate of peripheral compartment, *K*_*12*_ rate constant from compartment 1 to compartment 2, *K*_*21*_ rate constant from compartment 2 to compartment 1

#### PK/PD link model of parent SAX in T2DM rats

To study the true DPP-4 inhibition ratio of parent SAX in vivo, we substituted the plasma 5-OH SAX concentration after intragastric administration SAX into the above-mentioned established 5-OH SAX PK/PD model, and calculated the DPP-4 inhibition ratio produced by metabolite 5-OH SAX. It was subtracted from the total inhibition rate actually measured to obtain the actual inhibition ratio of the parent SAX. Use this inhibition ratio and the measured plasma SAX concentration to establish a PK/PD model of parent SAX.

**Fig. 8:**
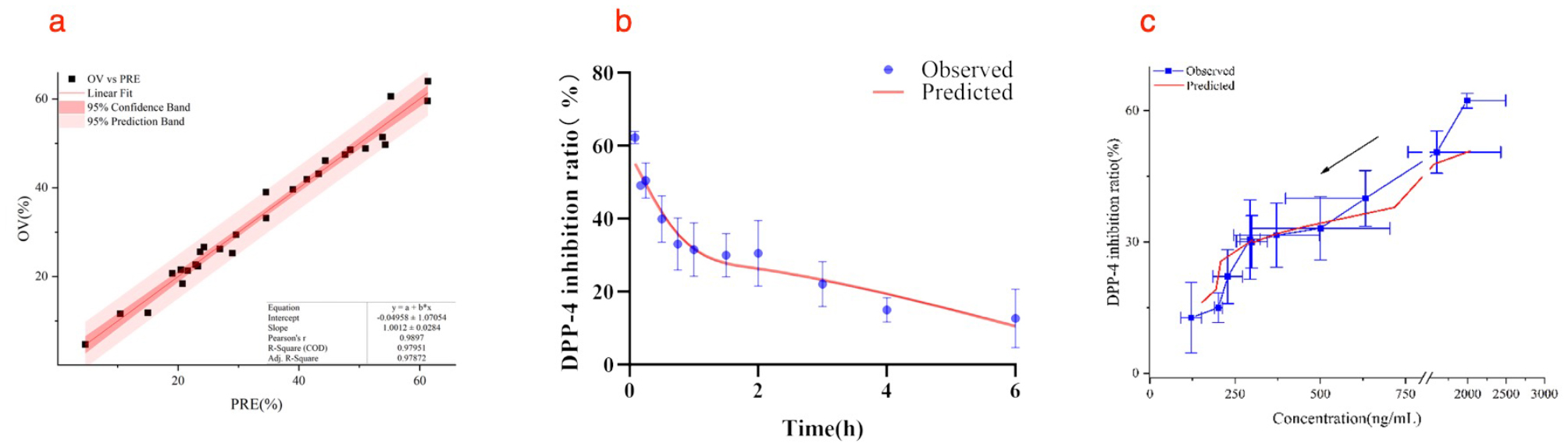
(**a**) Calculated SAX DPP-4 inhibition ratios versus predicted values. The linear line is red and the OV vs. PRE points are black solid square. (**b**) PD profiles of parent SAX in T2DM rats following a intragastric dose of 10 mg/kg. The blue solid points represent calculated individual DPP-4 inhibition ratios (*n*=3). The red solid line represents model fitting curve. (**c**) The dose-effect curve of parent SAX in T2DM rats. The arrow indicates that the efficacy of the drug shows a downward trend with the time of administration

Based on the established PK model parameters, the Hill model was used to fit the pharmacodynamic process of parent SAX in vivo. In the linear fitting curve plot (Fig. 8a), the linear correlation was good, and most of the observed values fell within the range of 95% CB and 95% PB. The observed and model-fitted DPP-4 inhibition ratio versus time course of SAX in T2DM rats is shown in Fig. 8b. The concentration-effect curve is shown in the Fig. 8c, the greater the plasma drug concentration, the higher the inhibition rate, and there is no hysteresis effect after administration. The estimated PD parameters are summarized in Table 12.

**Table 12:** Parameters of the PD model of parent SAX in T2DM rats (*n* = 3)

Parameter	Units	Mean	SD	CV
EC_50_	ng/mL	544.74	374.29	68.71
Gam		1.38	1.08	77.98
E_max_		71.47	25.21	35.27
stdev0		2.01	0.95	47.57

*EC*_*50*_ median effective concentration, *Gam* slope parameter of S-curve, *E*_*max*_ maximum effect of drug, *stdev0* fitting residual error

#### DPP-4 inhibition ratio, glucose, and insulin

**Fig. 9:**
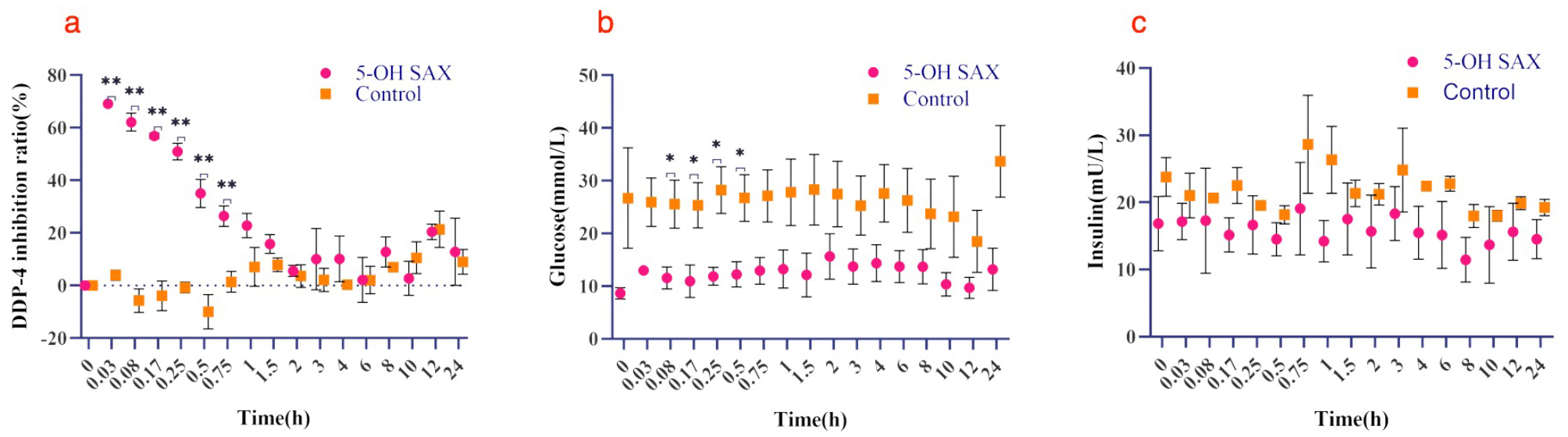
A scatter plot of observed values over time for each indicator after tail intravenous injection of 0.5 mg/kg 5-OH SAX (5-OH SAX groups) or saline (control groups) in T2DM rats. (**a**) DPP-4 inhibition ratio, (**b**) blood glucose and (**c**) insulin. Data are shown as mean ± SD (*n* = 3). *P < 0.05, **P < 0.01

Figure 9a shows that after T2DM rats tail vein injection of 5-OH SAX (0.5 mg/kg), the DPP-4 inhibition ratio decreased over time after reaching its largest (0.03 h) and stabilized after 2 h. Blood glucose (Fig. 9b) and insulin (Fig. 9c) change are stable over time. Compared to the control group, there were significant differences at 0.03, 0.08, 0.17, 0.25, 0.5, 0.75 h (*P*<0.05) for DPP-4 inhibition ratio; 0.08, 0.17, 0.25, and 0.5 h for glucose (*P*<0.05) and no significant differences for insulin within the sampling range.

**Fig. 10:**
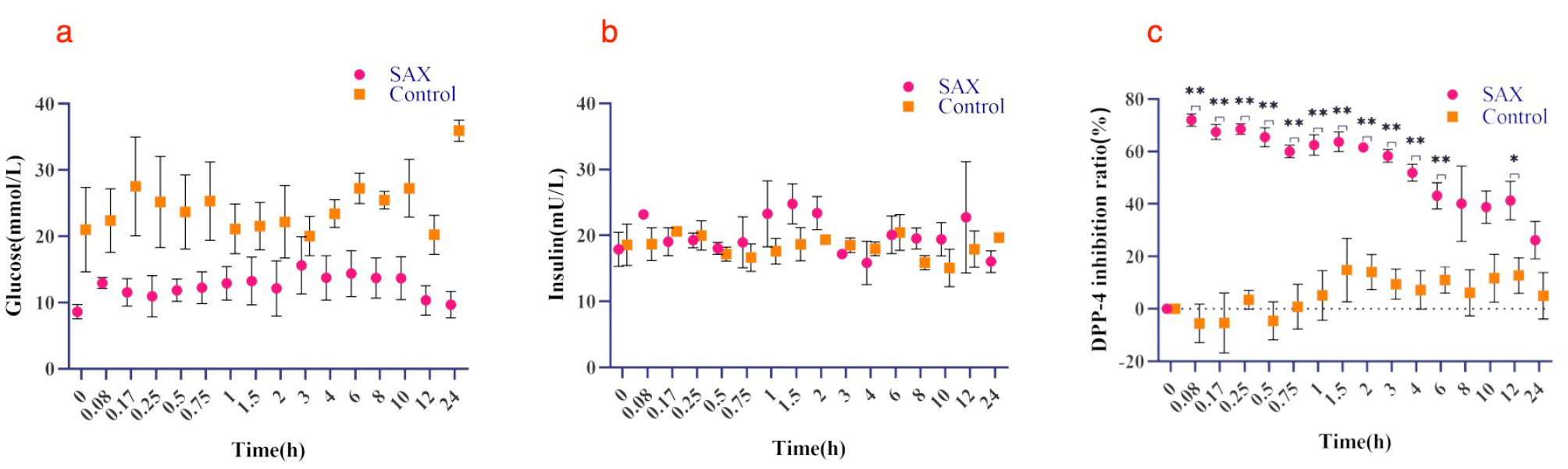
A scatter plot of observed values over time for each indicator after intragastric administration of 10 mg/kg SAX(SAX group)or 0.5% CMC-Na (control group) in T2DM rats. (**a**) DPP-4 inhibition ratio, (**b**) blood glucose and (**c**) insulin. Data are shown as mean ± SD (*n* = 3). *P < 0.05, **P < 0.01

It is different from the 5-OH SAX group, when administration of SAX (10 mg/kg), DPP-4 inhibition ratio stabilized after reaching the largest (0.08 h) and began to decrease at 3 h (Fig. 10a). Blood glucose (Fig. 10b) and insulin (Fig. 10c) change are also stable over time. Compared to the control group, there are significant differences at 0.08, 0.17, 0.25, 0.5, 0.75, 1, 1.5, 2, 3, 4, 6 h (*P* < 0.01) and 12 h (*P* < 0.05) for DPP-4 inhibition ratio, and no significant differences for glucose and insulin.

**Fig. 11:**
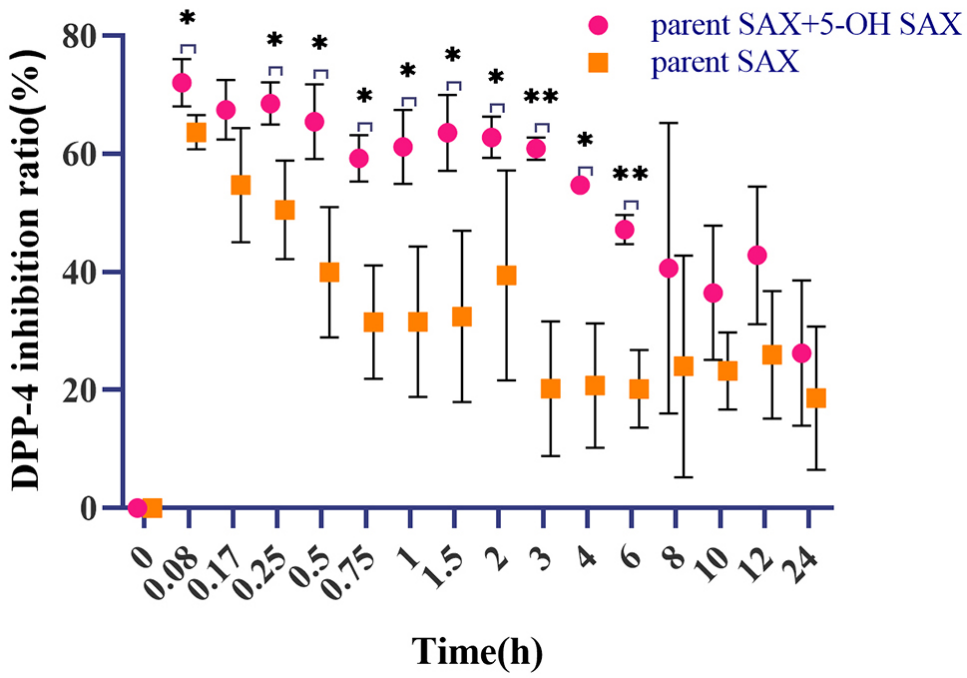
A scatter plot of observed total DPP-4 inhibition ratios and parent SAX DPP-4 inhibition ratios after intragastric administration of 10 mg/kg SAX in T2DM rats. Data are shown as mean ± SD (*n* = 3)

Figure 11 shows that after intragastric administration of SAX, there were statistical difference in plasma DPP-4 inhibition ratio compared with the inhibition rate produced by the parent SAX at 0.08, 0.25, 0.5, 0.75, 1, 1.5, 2, 3, 4, 6 h (*P* < 0.05, where in 3 h and 6 h *P* < 0.01). It can be seen from this that the DPP-4 inhibition effect of the metabolite 5-OH SAX cannot be ignored.

## Discussion

Study had found that a single DPP-4 enzyme can hydrolyze X-proline p-nitroanilides (x = glycylproline, alanylproline, lysylproline, arginylproline, glutamylproline and aspartylproline) [18]. Among these substrates, we chose Gly-Pro-pNA based on the following reasons. First, It has good water solubility and stable toluene sulfonation form. Second, Gly-Pro-pNA has become the substrate of a kit for detecting DPP-4 activity in scientific trials [19–21]. Third, X-prolyl dipeptide aminopeptidase may have the ability to degrade peptide fragments containing a certain sequence, while Gly-Pro-pNA has the same sequence. Fourth, under the condition of pH 8.7 (37 °C), the content of pNA produced by hydrolysis was higher, which was consistent with our research (Fig. 4b), but when the pH was 8.5 and the temperature was 40 °C, the pNA content was the highest. To increase the detection sensitivity, this condition was selected for detection.

The PK-PD model is a comprehensive study of the pharmacokinetic process in vivo and the quantitative kinetic process of pharmacodynamics [22]. It is the integration of two different processes into a unity. Its essence is a conversion process between drug or metabolite concentrations in the body and effect. This model can help understand the law of drug effect with concentration and time. SAX is the only metabolized DPP-4 inhibitor, and its total inhibitory effect is equal to the superposition of SAX and the active metabolite 5-OH SAX. The in vitro study reported that the inhibitory effect of 5-OH SAX was half of the parent drug SAX [10], but with no confirmation from in vivo studies. This study explores the relationship between the concentration of the parent drug SAX in the body and its actual efficacy. As an active metabolite, 5-OH SAX directly takes part in the circulation in the body which is better for intravenous injection. Plasma samples were collected after administration and at different time points to determine drug concentration, DPP-4 inhibition ratio, insulin and blood glucose. The concentrations and DPP-4 inhibition ratios were used to establish a PK/PD link model. Subsequently, we brought the 5-OH SAX plasma concentration collected after intragastric SAX into the established 5-OH SAX PK/PD model to get the inhibition rate of 5-OH SAX (In_5OHSAX_) at the corresponding concentration. Then the total inhibition rate (In_SAX+5OHSAX_) subtracts the In_5-OHSAX_, gives the parent SAX inhibition rate (In_SAX_), In_SAX_=In_SAX+5OHSAX_-In_5OHSAX_. Finally, the PK/PD model was fitted using the SAX blood concentration measured after intragastric administration and In_SAX_. After fitting different models, it was found that whether SAX or 5-OH SAX, the two-compartment additive model was the most suitable, and the goodness of fit and accuracy were better than other models. For intravenous injection of 5-OH SAX, based on its mechanism of action, the sigmoidal E_max_ model with E_0_ could describe the concentration-effect relationship. After intravenous administration of 5-OH SAX, the plasma concentration increased rapidly, 5-OH SAX and DPP-4 quickly combined, immediately produced inhibitory effect, and there was no hysteresis effect. For intragastric SAX, considering its absorption process, it was considered that there is a hysteresis effect, but in the model fitting process. It is found that the sigmoidal E_max_ without hysteresis effect was the most suitable to describe the PD process of the parent SAX, which was also consistent with its fast absorption feature [23, 24]. The absorption half-life of this study was 0.07 h. According to the final model fitting parameter EC_50_, EC_50, 5-OH SAX_=0.46EC_50, SAX(parent)_, it was believed that the inhibitory effect of 5-OH SAX was about half of the parent drug SAX, which is consistent with the literature [10].

The common clinical single dose of SAX is 5 mg/d, and the maximum inhibition rate of DPP-4 is 70% [8]. Through the pre-test, it was believed that 10 mg/kg can obtain an ideal inhibition rate effect.

It is reported that about 30% of SAX and 50% of 5-OH SAX in the human body are combined with DPP-4 after administration [25]. When the free drug in the body is eliminated, the combined drug will be released into the blood, and play a role. In addition, SAX and 5-OH SAX have a prolong dissociation rate from DPP-4 [10]. Although the experimental results of intragastric SAX (6.13 h) and intravenous injection of 5-OH SAX (0.36 h) have a shorter half-life, gavage SAX 10 mg/kg, the total inhibition rate for 24 h is 26%, and the parent SAX inhibition rate is 19% (Fig. 10b), which is consistent with the view that SAX can be maintained for 24 h after a single dose of SAX of 5 mg/d [26].

## Conclusion

A two-compartment PK model with first order absorption and PK/PD model with E_max_ and sigmoid were used to describe the relationship of parent SAX-induced DPP-4 inhibitory to its concentration in T2DM rats’ body. This model may predict the PK or PD effects of prodrug SAX in some special conditions, such as liver dysfunction, kidney dysfunction or DDIs. The limitation of this study is that the number of T2DM rats is small, resulting in a slightly larger coefficient of variation.

### Electronic supplementary material

Below is the link to the electronic supplementary material.

Supplementary Material 1

## Data Availability

All data generated or analyzed during this study are included in this published article and its supplementary information files.

## Abbreviations

A_a_: The amounts of SAX in the absorption compartment
A_1_: The amounts of SAX/5-OH SAX in the central compartment
A_2_: The amounts of SAX/5-OH SAX in the peripheral compartment
AUC: Area under the curve
C: The concentration of SAX/5-OH
C_max_: Maximum plasma concentration
CB: Confidence band
CE: Collision energy
CL: Clearance
CL_2_: The clearance rate from central compartment to peripheral compartment
CMC-Na: Sodium carboxymethyl cellulose
CV: Coefficient of variation
CYP3A4/5: Cytochrome P450 3A4/5
DM: Diabetes mellitus
DP: De-clustering Potential
DPP-4: Dipeptidyl peptidase IV
E_0_: Basic effect
E_max_: Maximum effect
EC_50_: Median effective concentration
ESI: Electrospray ionization
Gly-Pro-pNA: Glycyl-prolyl-p-nitroaniline
HDQC: High dilution quality control
HQC: High quality control
IACUC: Institutional Animal Care and Use Committee
In_5OHSAX_: Inhibition rate of 5-OH SAX
In_SAX_: Inhibition rate of parent SAX
In_SAX+5OHSAX_: The total inhibition rate
K_a_: The first order absorption rate
K_12_: Rate constant from compartment 1 to compartment 2
K_21_: Rate constant from compartment 2 to compartment 1
LDQC: Low dilution quality control
LLOQ: Lower limit of quantitation
LQC: Low quality control
MQC: Medium quality control
MRM: Multiple-reaction monitoring
OD: Optical density
OV: Observed values
PB: Prediction band
PK-PD: Pharmacokinetic-Pharmacodynamic
PRE: Predicted values
QC: Quality control samples
R^2^: Correlation coefficients
SAX: Saxagliptin
SD, %: Standard Deviation
SD: Sprague-Dawley
stdev0: Fitting residual error
T_max_: Time to maximum plasma concentration
t_1/2α_: Distribution phase half-life
t_1/2β_: Elimination half-life
T2DM: Type 2 Diabetes mellitus
UPLC-MS/MS: Ultra Performance Liquid Chromatography-Mass Spectrometry
V: The apparent volume of distribution
V_2_: The apparent distribution volume of peripheral compartment
VIL: Vildagliptin
5-OH SAX: 5-hydroxy Saxagliptin
γ: Gam, slope parameter of S-curve
