# Supplementary material for: Pharmacokinetic/Pharmacodynamic modelling of Saxagliptin and its active metabolite, 5-hydroxy Saxagliptin in rats with Type 2 Diabetes Mellitus
Source: BMC Pharmacol Toxicol. 2024 Jun 26;25:35. doi: 10.1186/s40360-024-00757-3 (PMC11299271; doi:10.1186/s40360-024-00757-3)
Supplement: Supplementary file 1 — Supplementary Material 1 [file 40360_2024_757_MOESM1_ESM.pdf]

Table S1. Intra-day precision and accuracy of the UPLC-MS/MS method for SAX

				Precision				Accuracy			
Sample Name	Analyte Concentration (ng/mL)	Analyte Peak Area (counts)	IS Peak Area (counts)	Calculated Concentration (ng/mL)	Mean	SD	CV	Accuracy (%)	Mean	SD	CV
LL	5	1.42E+04	1.35E+05	5.26				105.20			
LL	5	1.43E+04	1.36E+05	5.26				105.20			
LL	5	1.31E+04	1.39E+05	4.75				95.00			
LL	5	1.31E+04	1.39E+05	4.75				95.00			
LL	5	1.44E+04	1.36E+05	5.29	5.06	0.29	5.63	105.80	101.24	5.70	5.63
L	15	4.07E+04	1.33E+05	14.56				97.07			
L	15	4.23E+04	1.31E+05	15.34				102.27			
L	15	4.20E+04	1.35E+05	14.79				98.60			
L	15	4.15E+04	1.36E+05	14.52				96.80			
L	15	4.37E+04	1.35E+05	15.38	14.92	0.42	2.79	102.53	99.45	2.78	2.79
M	1000	2.91E+06	1.35E+05	998.68				99.87			
M	1000	2.88E+06	1.35E+05	988.73				98.87			
M	1000	2.96E+06	1.34E+05	1023.05				102.31			
M	1000	2.85E+06	1.31E+05	1006.89				100.69			
M	1000	2.95E+06	1.36E+05	1005.63	1004.60	12.59	1.25	100.56	100.46	1.26	1.25
H	2000	5.98E+06	1.37E+05	2021.21				101.06			
H	2000	5.94E+06	1.41E+05	1950.74				97.54			
H	2000	5.94E+06	1.33E+05	2068.06				103.40			
H	2000	5.86E+06	1.37E+05	1980.65				99.03			
H	2000	5.86E+06	1.36E+05	1995.22	2003.18	44.32	2.21	99.76	100.16	2.22	2.21

Table S2. Intra-day precision and accuracy of the UPLC-MS/MS method for 5-OH SAX											
				Precision				Accuracy			
Sample Name	Analyte Concentration (ng/mL)	Analyte Peak Area (counts)	IS Peak Area (counts)	Calculated Concentration (ng/mL)	Mean	SD	CV	Accuracy (%)	Mean	SD	CV
LL	1	1.00E+03	1.32E+05	1.02				102.00			
LL	1	1.01E+03	1.33E+05	1.03				103.00			
LL	1	1.05E+03	1.36E+05	1.10				110.00			
LL	1	1.06E+03	1.35E+05	1.18				118.00			
LL	1	1.02E+03	1.36E+05	0.98	1.06	0.08	7.43	98.00	106.20	7.89	7.43
L	3	1.46E+03	1.33E+05	2.98				99.33			
L	3	1.49E+03	1.31E+05	3.20				106.67			
L	3	1.50E+03	1.35E+05	3.05				101.67			
L	3	1.48E+03	1.36E+05	2.92				97.33			
L	3	1.47E+03	1.35E+05	2.92	3.01	0.12	3.88	97.33	100.47	3.90	3.88
M	200	4.84E+04	1.35E+05	202.71				101.36			
M	200	4.74E+04	1.35E+05	198.45				99.23			
M	200	4.82E+04	1.39E+05	195.96				97.98			
M	200	4.75E+04	1.31E+05	205.05				102.53			
M	200	4.87E+04	1.36E+05	202.46	200.93	3.65	1.82	101.23	100.46	1.83	1.82
H	400	9.65E+04	1.36E+05	404.46				101.12			
H	400	9.59E+04	1.37E+05	398.97				99.74			
H	400	9.67E+04	1.36E+05	405.30				101.33			
H	400	9.52E+04	1.37E+05	396.03				99.01			
H	400	9.69E+04	1.36E+05	406.15	402.18	4.44	1.10	101.54	100.55	1.11	1.10

Table S3. Inter-day precision and accuracy of the UPLC-MS/MS method for SAX												
					Precision				Accuracy			
	Sample Name	Analyte Concentration (ng/mL)	Analyte Peak Area (counts)	IS Peak Area (counts)	Calculated Concentration (ng/mL)	Mean	SD	CV	Accuracy (%)	Mean	SD	CV
Day 1	LL	5	1.54E+04	1.57E+05	4.93				98.60			
Day 2	LL	5	1.91E+04	1.34E+05	5.03				100.60			
Day 3	LL	5	1.47E+04	1.29E+05	4.58				91.60			
Day 4	LL	5	1.51E+04	1.33E+05	4.86				97.20			
Day 5	LL	5	1.69E+04	1.35E+05	5.43	4.97	0.31	6.21	108.60	99.32	6.17	6.21
Day 1	L	15	4.05E+04	1.31E+05	14.70				98.00			
Day 2	L	15	4.04E+04	1.03E+05	14.38				95.87			
Day 3	L	15	4.58E+04	1.33E+05	15.40				102.67			
Day 4	L	15	4.59E+04	1.35E+05	15.54				103.60			
Day 5	L	15	4.27E+04	1.36E+05	14.64	14.93	0.51	3.40	97.60	99.55	3.39	3.40
Day 1	M	1000	2.91E+06	1.36E+05	992.02				99.20			
Day 2	M	1000	2.80E+06	1.03E+05	1019.29				101.93			
Day 3	M	1000	2.74E+06	1.31E+05	981.20				98.12			
Day 4	M	1000	2.88E+06	1.38E+05	984.26				98.43			
Day 5	M	1000	2.81E+06	1.34E+05	1022.62	999.88	19.68	1.97	102.26	99.99	1.97	1.97
Day 1	H	2000	5.72E+06	1.36E+05	1947.57				97.38			
Day 2	H	2000	5.75E+06	1.11E+05	1939.84				96.99			
Day 3	H	2000	5.89E+06	1.34E+05	2062.86				103.14			
Day 4	H	2000	5.79E+06	1.34E+05	2037.66				101.88			
Day 5	H	2000	5.65E+06	1.42E+05	1940.24	1985.63	59.74	3.01	97.01	99.28	2.99	3.01

Table S4. Inter-day precision and accuracy of the UPLC-MS/MS method for 5-OH SAX

					Precision				Accuracy			
	Sample Name	Analyte Concentration (ng/mL)	Analyte Peak Area (counts)	IS Peak Area (counts)	Calculated Concentration (ng/mL)	Mean	SD	CV	Accuracy (%)	Mean	SD	CV
Day 1	LL	1	1.00E+03	1.32E+05	1.02				102.00			
Day 2	LL	1	1.95E+03	1.35E+05	0.93				93.00			
Day 3	LL	1	9.10E+02	1.39E+05	1.18				118.00			
Day 4	LL	1	1.01E+03	1.53E+05	0.98				98.00			
Day 5	LL	1	8.57E+02	1.45E+05	1.04	1.03	0.09	9.11	104.00	103.00	9.38	9.11
Day 1	L	3	1.50E+03	1.35E+05	3.05				101.67			
Day 2	L	3	2.52E+03	1.32E+05	3.04				101.33			
Day 3	L	3	1.55E+03	1.33E+05	3.54				118.00			
Day 4	L	3	1.35E+03	1.35E+05	3.07				102.33			
Day 5	L	3	1.31E+03	1.39E+05	3.12	3.16	0.21	6.71	104.00	105.47	7.08	6.71
Day 1	M	200	4.84E+04	1.35E+05	202.71				101.36			
Day 2	M	200	5.82E+04	1.33E+05	193.27				96.64			
Day 3	M	200	5.89E+04	1.33E+05	203.17				101.59			
Day 4	M	200	5.13E+04	1.52E+05	205.23				102.62			
Day 5	M	200	5.28E+04	1.44E+05	214.50	203.78	7.57	3.71	107.25	101.89	3.78	3.71
Day 1	H	400	9.46E+04	1.37E+05	393.51				98.38			
Day 2	H	400	1.15E+05	1.34E+05	384.80				96.20			
Day 3	H	400	1.18E+05	1.37E+05	396.57				99.14			
Day 4	H	400	1.02E+05	1.45E+05	429.00				107.25			
Day 5	H	400	9.59E+04	1.42E+05	397.16	400.21	16.84	4.21	99.29	100.05	4.21	4.21

Table S5. Extraction recovery evaluation of SAX							
		Extracted spiked sample (A)	Post-extracted spiked sample (B)	Extraction recovery			
Sample Name	Analyte Concentration (ng/mL)	Analyte Peak Area (counts)	Analyte Peak Area (counts)	A/B	Mean	SD	CV
L	15	4.12E+04	5.05E+04	81.58%			
L	15	4.05E+04	5.15E+04	78.64%			
L	15	4.23E+04	4.82E+04	87.76%			
L	15	4.11E+04	4.91E+04	83.71%			
L	15	4.15E+04	5.11E+04	81.21%	82.58%	3.41%	4.13
M	1000	2.81E+06	3.00E+06	93.80%			
M	1000	2.88E+06	2.99E+06	96.45%			
M	1000	2.90E+06	3.12E+06	93.02%			
M	1000	2.84E+06	3.13E+06	90.71%			
M	1000	2.91E+06	3.14E+06	92.85%	93.37%	2.07%	2.22
H	2000	5.61E+06	5.97E+06	93.95%			
H	2000	5.49E+06	5.78E+06	94.98%			
H	2000	5.59E+06	6.03E+06	92.70%			
H	2000	5.48E+06	6.15E+06	89.11%			
H	2000	5.72E+06	6.08E+06	94.08%	92.96%	2.31%	2.48

Table S6. Extraction recovery evaluation of 5-OH SAX							
		Extracted spiked sample (A)	Post-extracted spiked sample (B)	Extraction recovery			
Sample Name	Analyte Concentration (ng/mL)	Analyte Peak Area (counts)	Analyte Peak Area (counts)	A/B	Mean	SD	CV
L	3	1.48E+03	1.51E+03	98.01%			
L	3	1.50E+03	1.51E+03	99.34%			
L	3	1.41E+03	1.53E+03	92.16%			
L	3	1.46E+03	1.49E+03	97.99%			
L	3	1.42E+03	1.52E+03	93.42%	96.18%	3.18%	3.30
M	200	4.83E+04	5.22E+04	92.53%			
M	200	4.76E+04	4.96E+04	95.97%			
M	200	4.71E+04	5.24E+04	89.89%			
M	200	4.87E+04	5.28E+04	92.23%			
M	200	4.79E+04	5.40E+04	88.70%	91.86%	2.80%	3.05
H	400	9.57E+04	1.02E+05	93.82%			
H	400	9.38E+04	1.01E+05	92.87%			
H	400	9.63E+04	1.09E+05	88.35%			
H	400	9.49E+04	1.11E+05	85.50%			
H	400	9.61E+04	1.12E+05	85.80%	89.27%	3.90%	4.37

Table S7. Extraction recovery evaluation of VIL						
	Extracted spiked sample (A)	Post-extracted spiked sample (B)	Extraction recovery			
Analyte Concentration (ng/mL)	Analyte Peak Area (counts)	Analyte Peak Area (counts)	A/B	Mean	SD	CV
150	1.33E+05	1.47E+05	90.48%			
150	1.35E+05	1.46E+05	92.47%			
150	1.41E+05	1.51E+05	93.38%			
150	1.39E+05	1.47E+05	94.56%			
150	1.36E+05	1.51E+05	90.07%	92.19%	1.91%	2.07

Table S8. Matrix effect evaluation of SAX													
			Post-extracted spiked sample		Samples spiked with H_2O								
Lots	Sample Name	Analyte Concentration (ng/mL)	Analyte Peak Area (counts) (A)	IS Peak Area (counts) (B)	Analyte Peak Area (counts) (C)	IS Peak Area (counts) (D)	Analyte Matrix Factor (E=A/C)	SD	CV	IS Matrix Factor (F=B/D)	Normalization Analyte Matrix Factor (E/F)	SD	CV
1	L	15	4.81E+04	1.53E+05	5.39E+04	1.71E+05	89.24%			89.47%	99.74%		
	L	15	4.79E+04	1.47E+05	5.21E+04	1.77E+05	91.94%			83.05%	110.70%		
	L	15	4.91E+04	1.52E+05	5.43E+04	1.73E+05	90.42%			87.86%	102.92%		
		Mean	4.84E+04	1.51E+05	5.34E+04	1.74E+05	90.53%	1.35%	1.49%	86.80%	104.45%	5.64%	5.40%
2	L	15	5.11E+04	1.47E+05	5.21E+04	1.81E+05	98.08%			81.22%	120.77%		
	L	15	4.89E+04	1.48E+05	5.11E+04	1.76E+05	95.69%			84.09%	113.80%		
	L	15	5.12E+04	1.45E+05	5.45E+04	1.73E+05	93.94%			83.82%	112.09%		
		Mean	5.04E+04	1.47E+05	5.26E+04	1.77E+05	95.91%	2.08%	2.16%	83.04%	115.55%	4.60%	3.98%
3	L	15	4.78E+04	1.42E+05	5.23E+04	1.71E+05	91.40%			83.04%	110.06%		
	L	15	4.92E+04	1.41E+05	5.21E+04	1.76E+05	94.43%			80.11%	117.87%		
	L	15	4.91E+04	1.48E+05	5.33E+04	1.75E+05	92.12%			84.57%	108.93%		
		Mean	4.87E+04	1.44E+05	5.26E+04	1.74E+05	92.65%	1.59%	1.71%	82.58%	112.29%	4.87%	4.34%
4	L	15	5.15E+04	1.46E+05	5.22E+04	1.74E+05	98.66%			83.91%	117.58%		
	L	15	5.11E+04	1.45E+05	5.33E+04	1.73E+05	95.87%			83.82%	114.39%		
	L	15	5.09E+04	1.50E+05	5.47E+04	1.81E+05	93.05%			82.87%	112.28%		
		Mean	5.12E+04	1.47E+05	5.34E+04	1.76E+05	95.86%	2.80%	2.92%	83.53%	114.75%	2.67%	2.32%
5	L	15	4.73E+04	1.48E+05	5.21E+04	1.74E+05	90.79%			85.06%	106.74%		
	L	15	4.77E+04	1.47E+05	5.19E+04	1.76E+05	91.91%			83.52%	110.04%		
	L	15	4.77E+04	1.53E+05	5.46E+04	1.82E+05	87.36%			84.07%	103.92%		
		Mean	4.76E+04	1.49E+05	5.29E+04	1.77E+05	90.02%	2.37%	2.63%	84.22%	106.90%	3.06%	2.86%
6	L	15	4.98E+04	1.47E+05	5.41E+04	1.71E+05	92.05%			85.96%	107.08%		
	L	15	4.67E+04	1.51E+05	5.29E+04	1.74E+05	88.28%			86.78%	101.73%		
	L	15	4.76E+04	1.55E+05	5.46E+04	1.75E+05	87.18%			88.57%	98.43%		
		Mean	4.80E+04	1.51E+05	5.39E+04	1.73E+05	89.17%	2.56%	2.87%	87.11%	102.41%	4.37%	4.26%

Table S8. Matrix effect evaluation of SAX

			Post-extracted spiked sample		Samples spiked with H_2O								
Lots	Sample Name	Analyte Concentration (ng/mL)	Analyte Peak Area (counts) (A)	IS Peak Area (counts) (B)	Analyte Peak Area (counts) (C)	IS Peak Area (counts) (D)	Analyte Matrix Factor (E=A/C)	SD	CV	IS Matrix Factor (F=B/D)	Normalization Analyte Matrix Factor (E/F)	SD	CV
		Lots											
		1	4.84E+04	1.51E+05	5.34E+04	1.74E+05	90.53%			86.80%	104.45%		
		2	5.04E+04	1.47E+05	5.26E+04	1.77E+05	95.91%			83.04%	115.55%		
		3	4.87E+04	1.44E+05	5.26E+04	1.74E+05	92.65%			82.58%	112.29%		
		4	5.12E+04	1.47E+05	5.34E+04	1.76E+05	95.86%			83.53%	114.75%		
		5	4.76E+04	1.49E+05	5.29E+04	1.77E+05	90.02%			84.22%	106.90%		
		6	4.80E+04	1.51E+05	5.39E+04	1.73E+05	89.17%			87.11%	102.41%		
		Mean	4.90E+04	1.48E+05	5.31E+04	1.75E+05	92.36%			84.54%	109.39%		
		SD	1.42E+03	2.81E+03	5.31E+02	1.71E+03	2.96%			1.94%	5.56%		
		CV	2.90%	1.90%	1.00%	0.98%	3.21%			2.30%	5.08%		
1	H	2000	5.83E+06	1.42E+05	6.90E+06	1.76E+05	84.49%			80.68%	104.72%		
	H	2000	5.78E+06	1.45E+05	6.95E+06	1.74E+05	83.17%			83.33%	99.80%		
	H	2000	6.03E+06	1.49E+05	7.00E+06	1.71E+05	86.14%			87.13%	98.86%		
		Mean	5.88E+06	1.45E+05	6.95E+06	1.74E+05	84.60%	1.49%	1.76%	83.72%	101.13%	3.15%	3.11%
2	H	2000	5.97E+06	1.43E+05	6.88E+06	1.70E+05	86.79%			84.12%	103.17%		
	H	2000	5.78E+06	1.44E+05	6.91E+06	1.73E+05	83.65%			83.24%	100.49%		
	H	2000	6.03E+06	1.48E+05	6.93E+06	1.75E+05	87.01%			84.57%	102.89%		
		Mean	5.93E+06	1.45E+05	6.91E+06	1.73E+05	85.82%	1.88%	2.19%	83.98%	102.18%	1.47%	1.44%
3	H	2000	5.83E+06	1.48E+05	6.92E+06	1.71E+05	84.25%			86.55%	97.34%		
	H	2000	5.65E+06	1.44E+05	6.81E+06	1.72E+05	82.97%			83.72%	99.10%		
	H	2000	5.78E+06	1.49E+05	6.98E+06	1.69E+05	82.81%			88.17%	93.92%		
		Mean	5.75E+06	1.47E+05	6.90E+06	1.71E+05	83.34%	0.79%	0.95%	86.15%	96.79%	2.63%	2.72%
4	H	2000	6.11E+06	1.45E+05	7.01E+06	1.79E+05	87.16%			81.01%	107.60%		
	H	2000	5.69E+06	1.44E+05	6.89E+06	1.83E+05	82.58%			78.69%	104.95%		
	H	2000	6.13E+06	1.46E+05	6.91E+06	1.79E+05	88.71%			81.56%	108.76%		
		Mean	5.98E+06	1.45E+05	6.94E+06	1.80E+05	86.15%	3.19%	3.70%	80.42%	107.10%	1.95%	1.82%

Table S8. Matrix effect evaluation of SAX

			Post-extracted spiked sample		Samples spiked with H_2O								
Lots	Sample Name	Analyte Concentration (ng/mL)	Analyte Peak Area (counts) (A)	IS Peak Area (counts) (B)	Analyte Peak Area (counts) (C)	IS Peak Area (counts) (D)	Analyte Matrix Factor (E=A/C)	SD	CV	IS Matrix Factor (F=B/D)	Normalization Analyte Matrix Factor (E/F)	SD	CV
5	H	2000	5.67E+06	1.52E+05	6.79E+06	1.74E+05	83.51%			87.36%	95.59%		
	H	2000	5.74E+06	1.49E+05	7.03E+06	1.79E+05	81.65%			83.24%	98.09%		
	H	2000	5.64E+06	1.51E+05	6.85E+06	1.66E+05	82.34%			90.96%	90.51%		
		Mean	5.68E+06	1.51E+05	6.89E+06	1.73E+05	82.50%	0.94%	1.14%	87.19%	94.73%	3.86%	4.07%
6	H	2000	5.83E+06	1.48E+05	6.90E+06	1.75E+05	84.49%			84.57%	99.91%		
	H	2000	5.78E+06	1.49E+05	6.95E+06	1.76E+05	83.17%			84.66%	98.24%		
	H	2000	5.73E+06	1.47E+05	7.00E+06	1.86E+05	81.86%			79.03%	103.57%		
		Mean	5.78E+06	1.48E+05	6.95E+06	1.79E+05	83.17%	1.32%	1.58%	82.75%	100.57%	2.73%	2.72%
		Lots											
		1	5.88E+06	1.45E+05	6.95E+06	1.74E+05	84.60%			83.72%	101.13%		
		2	5.93E+06	1.45E+05	6.91E+06	1.73E+05	85.82%			83.98%	102.18%		
		3	5.75E+06	1.47E+05	6.90E+06	1.71E+05	83.34%			86.15%	96.79%		
		4	5.98E+06	1.45E+05	6.94E+06	1.80E+05	86.15%			80.42%	107.10%		
		5	5.68E+06	1.51E+05	6.89E+06	1.73E+05	82.50%			87.19%	94.73%		
		6	5.78E+06	1.48E+05	6.95E+06	1.79E+05	83.17%			82.75%	100.57%		
		Mean	5.83E+06	1.47E+05	6.92E+06	1.75E+05	84.26%			84.03%	100.42%		
		SD	1.12E+05	2.24E+03	2.60E+04	3.86E+03	1.50%			2.42%	4.33%		
		CV	1.93%	1.52%	0.38%	2.21%	1.78%			2.88%	4.31%		

Table S9. Matrix effect evaluation of 5-OH SAX

			Post-extracted spiked sample		Samples spiked with H_2O								
Lots	Sample Name	Analyte Concentration (ng/mL)	Analyte Peak Area (counts) (A)	IS Peak Area (counts) (B)	Analyte Peak Area (counts) (C)	IS Peak Area (counts) (D)	Analyte Matrix Factor (E=A/C)	SD	CV	IS Matrix Factor(F=B/D)	Normalization Analyte Matrix Factor (E/F)	SD	CV
1	L	3	1.50E+03	1.51E+05	1.72E+03	1.79E+05	87.21%			84.36%	103.38%		
	L	3	1.52E+03	1.49E+05	1.70E+03	1.76E+05	89.41%			84.66%	105.61%		
	L	3	1.49E+03	1.45E+05	1.66E+03	1.73E+05	89.76%			83.82%	107.09%		
		Mean	1.50E+03	1.48E+05	1.69E+03	1.76E+05	88.79%	1.38%	1.56%	84.28%	105.36%	1.87%	1.77%
2	L	3	1.51E+03	1.47E+05	1.69E+03	1.79E+05	89.35%			82.12%	108.80%		
	L	3	1.50E+03	1.50E+05	1.61E+03	1.77E+05	93.17%			84.75%	109.94%		
	L	3	1.53E+03	1.45E+05	1.73E+03	1.74E+05	88.44%			83.33%	106.13%		
		Mean	1.51E+03	1.47E+05	1.68E+03	1.77E+05	90.32%	2.51%	2.78%	83.40%	108.29%	1.96%	1.81%
3	L	3	1.45E+03	1.49E+05	1.75E+03	1.74E+05	82.86%			85.63%	96.76%		
	L	3	1.47E+03	1.41E+05	1.71E+03	1.75E+05	85.96%			80.57%	106.69%		
	L	3	1.48E+03	1.53E+05	1.78E+03	1.73E+05	83.15%			88.44%	94.01%		
		Mean	1.47E+03	1.48E+05	1.75E+03	1.74E+05	83.99%	1.72%	2.04%	84.88%	99.16%	6.67%	6.73%
4	L	3	1.45E+03	1.49E+05	1.75E+03	1.74E+05	82.86%			85.63%	96.76%		
	L	3	1.49E+03	1.45E+05	1.74E+03	1.75E+05	85.63%			82.86%	103.35%		
	L	3	1.43E+03	1.51E+05	1.86E+03	1.73E+05	76.88%			87.28%	88.08%		
		Mean	1.46E+03	1.48E+05	1.78E+03	1.74E+05	81.79%	4.47%	5.47%	85.26%	96.06%	7.66%	7.97%
5	L	3	1.48E+03	1.44E+05	1.81E+03	1.84E+05	81.77%			78.26%	104.48%		
	L	3	1.52E+03	1.48E+05	1.69E+03	1.79E+05	89.94%			82.68%	108.78%		
	L	3	1.49E+03	1.47E+05	1.74E+03	1.76E+05	85.63%			83.52%	102.53%		
		Mean	1.50E+03	1.46E+05	1.75E+03	1.80E+05	85.78%	4.09%	4.77%	81.49%	105.26%	3.20%	3.04%
6	L	3	1.49E+03	1.46E+05	1.72E+03	1.79E+05	86.63%			81.56%	106.21%		
	L	3	1.52E+03	1.41E+05	1.70E+03	1.77E+05	89.41%			79.66%	112.24%		
	L	3	1.51E+03	1.49E+05	1.66E+03	1.82E+05	90.96%			81.87%	111.11%		
		Mean	1.51E+03	1.45E+05	1.69E+03	1.79E+05	89.00%	2.20%	2.47%	81.03%	109.85%	3.21%	2.92%

Table S9. Matrix effect evaluation of 5-OH SAX

			Post-extracted spiked sample		Samples spiked with H_2O								
Lots	Sample Name	Analyte Concentration (ng/mL)	Analyte Peak Area (counts) (A)	IS Peak Area (counts) (B)	Analyte Peak Area (counts) (C)	IS Peak Area (counts) (D)	Analyte Matrix Factor (E=A/C)	SD	CV	IS Matrix Factor(F=B/D)	Normalization Analyte Matrix Factor (E/F)	SD	CV
		Lots											
		1	1.50E+03	1.48E+05	1.69E+03	1.76E+05	88.79%			84.28%	105.36%		
		2	1.51E+03	1.47E+05	1.68E+03	1.77E+05	90.32%			83.40%	108.29%		
		3	1.47E+03	1.48E+05	1.75E+03	1.74E+05	83.99%			84.88%	99.16%		
		4	1.46E+03	1.48E+05	1.78E+03	1.74E+05	81.79%			85.26%	96.06%		
		5	1.50E+03	1.46E+05	1.75E+03	1.80E+05	85.78%			81.49%	105.26%		
		6	1.51E+03	1.45E+05	1.69E+03	1.79E+05	89.00%			81.03%	109.85%		
		Mean	1.49E+03	1.47E+05	1.72E+03	1.77E+05	86.61%			83.39%	104.00%		
		SD	2.32E+01	1.19E+03	4.16E+01	2.48E+03	3.32%			1.77%	5.34%		
		CV	1.56%	0.81%	2.42%	1.40%	3.83%			2.12%	5.13%		
1	H	400	9.90E+04	1.43E+05	1.12E+05	1.76E+05	88.39%			81.25%	108.79%		
	H	400	1.01E+05	1.45E+05	1.21E+05	1.74E+05	83.47%			83.33%	100.17%		
	H	400	9.80E+04	1.47E+05	1.23E+05	1.71E+05	79.67%			85.96%	92.68%		
		Mean	9.93E+04	1.45E+05	1.19E+05	1.74E+05	83.85%	4.37%	5.21%	83.52%	100.55%	8.06%	8.02%
2	H	400	1.02E+05	1.43E+05	1.25E+05	1.77E+05	81.60%			80.79%	101.00%		
	H	400	1.01E+05	1.47E+05	1.19E+05	1.70E+05	84.87%			86.47%	98.15%		
	H	400	1.08E+05	1.51E+05	1.18E+05	1.79E+05	91.53%			84.36%	108.50%		
		Mean	1.04E+05	1.47E+05	1.21E+05	1.75E+05	86.00%	5.06%	5.88%	83.87%	102.55%	5.34%	5.21%
3	H	400	9.40E+04	1.49E+05	1.19E+05	1.75E+05	78.99%			85.14%	92.78%		
	H	400	1.01E+05	1.48E+05	1.31E+05	1.74E+05	77.10%			85.06%	90.64%		
	H	400	9.90E+04	1.41E+05	1.14E+05	1.76E+05	86.84%			80.11%	108.40%		
		Mean	9.80E+04	1.46E+05	1.21E+05	1.75E+05	80.98%	5.17%	6.38%	83.44%	97.27%	9.69%	9.97%
4	H	400	9.80E+04	1.40E+05	1.09E+05	1.73E+05	89.91%			80.92%	111.10%		
	H	400	1.01E+05	1.42E+05	1.15E+05	1.71E+05	87.83%			83.04%	105.76%		
	H	400	1.12E+05	1.43E+05	1.33E+05	1.70E+05	84.21%			84.12%	100.11%		
		Mean	1.04E+05	1.42E+05	1.19E+05	1.71E+05	87.31%	2.88%	3.30%	82.69%	105.66%	5.50%	5.20%
5	H	400	9.80E+04	1.39E+05	1.18E+05	1.71E+05	83.05%			81.29%	102.17%		

Table S9. Matrix effect evaluation of 5-OH SAX													
			Post-extracted spiked sample		Samples spiked with H_2O								
Lots	Sample Name	Analyte Concentration (ng/mL)	Analyte Peak Area (counts) (A)	IS Peak Area (counts) (B)	Analyte Peak Area (counts) (C)	IS Peak Area (counts) (D)	Analyte Matrix Factor (E=A/C)	SD	CV	IS Matrix Factor(F=B/D)	Normalization Analyte Matrix Factor (E/F)	SD	CV
	H	400	9.90E+04	1.42E+05	1.19E+05	1.73E+05	83.19%			82.08%	101.36%		
	H	400	9.80E+04	1.44E+05	1.25E+05	1.70E+05	78.40%			84.71%	92.56%		
		Mean	9.83E+04	1.42E+05	1.21E+05	1.71E+05	81.55%	2.73%	3.34%	82.69%	98.69%	5.33%	5.40%
6	H	400	1.11E+05	1.46E+05	1.22E+05	1.73E+05	90.98%			84.39%	107.81%		
	H	400	1.09E+05	1.45E+05	1.18E+05	1.75E+05	92.37%			82.86%	111.48%		
	H	400	1.15E+05	1.45E+05	1.31E+05	1.73E+05	87.79%			83.82%	104.74%		
		Mean	1.12E+05	1.45E+05	1.24E+05	1.74E+05	90.38%	2.35%	2.60%	83.69%	108.01%	3.38%	3.13%
		Lots											
		1	9.93E+04	1.45E+05	1.19E+05	1.74E+05	83.85%			83.52%	100.55%		
		2	1.04E+05	1.47E+05	1.21E+05	1.75E+05	86.00%			83.87%	102.55%		
		3	9.80E+04	1.46E+05	1.21E+05	1.75E+05	80.98%			83.44%	97.27%		
		4	1.04E+05	1.42E+05	1.19E+05	1.71E+05	87.31%			82.69%	105.66%		
		5	9.83E+04	1.42E+05	1.21E+05	1.71E+05	81.55%			82.69%	98.69%		
		6	1.12E+05	1.45E+05	1.24E+05	1.74E+05	90.38%			83.69%	108.01%		
		Mean	1.02E+05	1.44E+05	1.21E+05	1.73E+05	85.01%			83.32%	102.12%		
		SD	5.18E+03	2.26E+03	1.80E+03	1.73E+03	3.60%			0.51%	4.13%		
		CV	5.06%	1.56%	1.49%	1.00%	4.23%			0.61%	4.04%		

Table S10. Stability evaluation of the UPLC-MS/MS method for SAX

					Precision	Accuracy			
Sample Condition	Sample Name	Analyte Concentration (ng/mL)	Analyte Peak Area (counts)	IS Peak Area (counts)	Calculated Concentration (ng/mL)	Accuracy (%)	Mean	SD	CV
Freshly Preparations	L	15	4.07E+04	1.33E+05	14.56	97.07			
	L	15	4.23E+04	1.31E+05	15.34	102.27			
	L	15	4.37E+04	1.35E+05	15.38	102.53	100.62	3.08	3.06
	H	2000	5.98E+06	1.37E+05	2021.21	101.06			
	H	2000	5.86E+06	1.37E+05	1980.65	99.03			
	H	2000	5.69E+06	1.36E+05	1937.35	96.87	98.99	2.10	2.12
Freeze-thaw three cycles	L	15	4.20E+04	1.35E+05	14.79	98.60			
	L	15	4.15E+04	1.36E+05	14.52	96.80			
	L	15	4.12E+04	1.38E+05	14.21	94.73	96.71	1.93	2.00
	H	2000	5.73E+06	1.41E+05	1881.79	94.09			
	H	2000	5.55E+06	1.33E+05	1932.30	96.62			
	H	2000	5.77E+06	1.38E+05	1936.11	96.81	95.84	1.52	1.58
Room temperature for 4 h	L	15	4.23E+04	1.33E+05	15.11	100.73			
	L	15	4.09E+04	1.34E+05	14.52	96.80			
	L	15	4.24E+04	1.35E+05	14.93	99.53	99.02	2.02	2.04
	H	2000	5.84E+06	1.39E+05	1945.50	97.28			
	H	2000	5.95E+06	1.33E+05	2071.54	103.58			
	H	2000	5.71E+06	1.35E+05	1958.55	97.93	99.59	3.47	3.48

Table S10. Stability evaluation of the UPLC-MS/MS method for SAX									
					Precision	Accuracy			
4℃ for 12 h	L	15	4.24E+04	1.33E+05	15.15	101.00			
	L	15	4.28E+04	1.32E+05	15.40	102.67			
	L	15	4.21E+04	1.36E+05	14.72	98.13	100.60	2.29	2.28
	H	2000	5.82E+06	1.33E+05	2026.29	101.31			
	H	2000	5.62E+06	1.32E+05	1971.49	98.57			
	H	2000	5.51E+06	1.31E+05	1947.66	97.38	99.09	2.02	2.03
- 80℃ for 7 days	L	15	4.27E+04	1.34E+05	15.14	100.93			
	L	15	4.17E+04	1.35E+05	14.69	97.93			
	L	15	4.18E+04	1.37E+05	14.52	96.80	98.56	2.14	2.17
	H	2000	5.87E+06	1.39E+05	1955.49	97.77			
	H	2000	5.76E+06	1.33E+05	2005.40	100.27			
	H	2000	5.69E+06	1.37E+05	1923.21	96.16	98.07	2.07	2.11
autosampler at 4℃ for up to 16 h	L	15	4.25E+04	1.33E+05	15.18	101.20			
	L	15	4.11E+04	1.34E+05	14.59	97.27			
	L	15	4.20E+04	1.35E+05	14.79	98.60	99.02	2.00	2.02
	H	2000	5.59E+06	1.35E+05	1917.40	95.87			
	H	2000	5.74E+06	1.33E+05	1998.44	99.92			
	H	2000	5.82E+06	1.37E+05	1967.14	98.36	98.05	2.04	2.08

Table S11. Stability evaluation of the UPLC-MS/MS method for 5-OH SAX									
					Precision	Accuracy			
Sample Condition	Sample Name	Analyte Concentration (ng/mL)	Analyte Peak Area (counts)	IS Peak Area (counts)	Calculated Concentration (ng/mL)	Accuracy (%)	Mean	SD	CV
Freshly Preparations	L	3	1.46E+03	1.33E+05	2.98	99.33			
	L	3	1.48E+03	1.31E+05	3.16	105.33			
	L	3	1.47E+03	1.35E+05	2.92	97.33	100.67	4.16	4.14
	H	400	9.67E+04	1.35E+05	408.33	102.08			
	H	400	9.42E+04	1.37E+05	391.83	97.96			
	H	400	9.69E+04	1.34E+05	412.26	103.07	101.04	2.71	2.68
Freeze-thaw three cycles	L	3	1.47E+03	1.35E+05	2.92	97.33			
	L	3	1.44E+03	1.36E+05	2.75	91.67			
	L	3	1.49E+03	1.38E+05	2.87	95.67	94.89	2.91	3.07
	H	400	9.45E+04	1.37E+05	393.09	98.27			
	H	400	9.18E+04	1.38E+05	378.98	94.75			
	H	400	9.01E+04	1.36E+05	377.41	94.35	95.79	2.16	2.25
Room temperature for 4 h	L	3	1.50E+03	1.35E+05	3.05	101.67			
	L	3	1.48E+03	1.34E+05	3.01	100.33			
	L	3	1.49E+03	1.37E+05	2.92	97.33	99.78	2.22	2.22
	H	400	9.45E+04	1.34E+05	401.97	100.49			
	H	400	9.18E+04	1.36E+05	384.60	96.15			
	H	400	9.23E+04	1.33E+05	395.51	98.88	98.51	2.19	2.23

Table S11. Stability evaluation of the UPLC-MS/MS method for 5-OH SAX

					Precision	Accuracy			
4℃ for 12 h	L	3	1.44E+03	1.32E+05	2.94	98.00			
	L	3	1.45E+03	1.31E+05	3.03	101.00			
	L	3	1.52E+03	1.36E+05	3.09	103.00	100.67	2.52	2.50
	H	400	9.57E+04	1.35E+05	404.07	101.02			
	H	400	9.19E+04	1.36E+05	385.02	96.26			
	H	400	9.68E+04	1.37E+05	402.74	100.69	99.32	2.66	2.68
- 80℃ for 7 days	L	3	1.49E+03	1.35E+05	3.01	100.33			
	L	3	1.39E+03	1.31E+05	2.76	92.00			
	L	3	1.45E+03	1.36E+05	2.79	93.00	95.11	4.55	4.78
	H	400	9.43E+04	1.35E+05	398.11	99.53			
	H	400	9.38E+04	1.36E+05	393.05	98.26			
	H	400	9.24E+04	1.39E+05	378.71	94.68	97.49	2.52	2.58
autosampler at 4℃ for up to 16 h	L	3	1.51E+03	1.35E+05	3.09	103.00			
	L	3	1.52E+03	1.37E+05	3.04	101.33			
	L	3	1.50E+03	1.38E+05	2.91	97.00	100.44	3.10	3.08
	H	400	9.51E+04	1.37E+05	395.61	98.90			
	H	400	9.42E+04	1.33E+05	403.72	100.93			
	H	400	9.76E+04	1.34E+05	415.26	103.82	101.22	2.47	2.44

Table S12. Dilution Integrity analyses of the UPLC-MS/MS method for simultaneous determination of SAX and 5-OH SAX

						Precision					Accuracy			
Analytes	Sample Name	Dilution factor	Analyte Concentration (ng/mL)	Analyte Peak Area (counts)	IS Peak Area (counts)	Calculated Concentration (ng/mL)		Mean	SD	CV	Accuracy (%)	Mean	SD	CV
SAX	LDQC	2	2500	3.59E+06	1.36E+05	2444.95	2444.95				97.80			
	LDQC	2	2500	3.60E+06	1.35E+05	2469.92	2469.92				98.80			
	LDQC	2	2500	3.80E+06	1.37E+05	2565.66	2565.66				102.63			
	LDQC	2	2500	3.68E+06	1.33E+05	2561.35	2561.35				102.45			
	LDQC	2	2500	3.66E+06	1.38E+05	2458.51	2458.51	2500.08	58.59	2.34	98.34	100.00	2.34	2.34
	HDQC	2	3500	5.04E+06	1.37E+05	3407.11	3407.11				97.35			
	HDQC	2	3500	5.34E+06	1.38E+05	3580.36	3580.36				102.30			
	HDQC	2	3500	5.14E+06	1.35E+05	3526.16	3526.16				100.75			
	HDQC	2	3500	5.29E+06	1.37E+05	3576.07	3576.07				102.17			
	HDQC	2	3500	5.01E+06	1.36E+05	3411.73	3411.73	3500.28	85.66	2.45	97.48	100.01	2.45	2.45
5-OH SAX	LDQC	8	500	1.61E+04	1.34E+05	525.74	525.74				105.15			
	LDQC	8	500	1.51E+04	1.36E+05	483.81	483.81				96.76			
	LDQC	8	500	1.60E+04	1.38E+05	506.40	506.40				101.28			
	LDQC	8	500	1.57E+04	1.33E+05	516.07	516.07				103.21			
	LDQC	8	500	1.52E+04	1.37E+05	483.44	483.44	503.09	19.04	3.78	96.69	100.62	3.81	3.78
	HDQC	8	3500	1.03E+05	1.38E+05	3398.29	3398.29				97.09			
	HDQC	8	3500	1.10E+05	1.35E+05	3723.01	3723.01				106.37			
	HDQC	8	3500	1.01E+05	1.34E+05	3442.20	3442.20				98.35			
	HDQC	8	3500	1.02E+05	1.35E+05	3443.75	3443.75				98.39			
	HDQC	8	3500	1.10E+05	1.38E+05	3641.50	3641.50	3529.75	143.34	4.06	104.04	100.85	4.10	4.06

Table S13. The data of Plasma pNA calibration curves

Nominal concentration (mg/mL)	Line1	Line2	Line3	Line4	Line5	Mean
0.0625	0.0850	0.0715	0.0725	0.0685	0.0620	0.0719
0.125	0.1575	0.1400	0.1490	0.1410	0.1190	0.1413
0.25	0.3060	0.2950	0.2825	0.2770	0.2565	0.2834
0.5	0.6030	0.5730	0.5895	0.5415	0.5435	0.5701
0.75	0.8850	0.8425	0.8780	0.8515	0.7725	0.8459
1	1.1325	1.1045	1.1560	1.1040	1.0175	1.1029

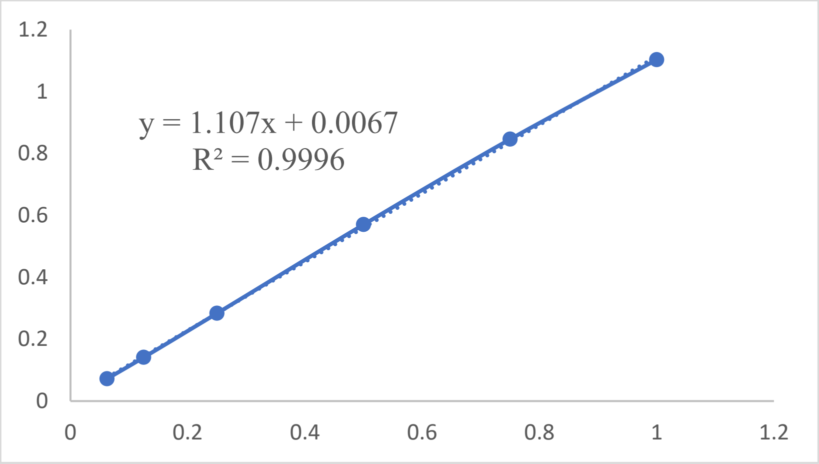

Fig. S1 The calibration curve of mean Plasma pNA

Table S14. Intra-day and inter-day precision and accuracy of the plasma pNA																
Intra-day																
	Mearsurment concentration(mg/mL)								Accuary(%)							
	Precision								Accuary(%)							
Nominal concentration (mg/mL)	Batch1	Batch2	Batch3	Batch4	Batch5	Mean	SD	CV	Batch1	Batch2	Batch3	Batch4	Batch5	Mean	SD	CV
0.0625	0.07	0.05	0.06	0.06	0.06	0.06	0.01	9.92	108.11	81.50	97.06	98.94	97.06	96.53	9.57	9.92
0.125	0.12	0.12	0.13	0.12	0.12	0.12	0.01	4.58	99.92	93.19	105.20	95.99	98.81	98.62	4.51	4.58
0.25	0.24	0.24	0.25	0.28	0.22	0.25	0.02	8.43	97.13	96.55	98.89	111.22	88.12	98.38	8.30	8.43
0.75	0.67	0.78	0.79	0.63	0.75	0.73	0.07	9.45	89.93	104.23	104.80	84.36	100.41	96.74	9.14	9.45
Inter-day																
	Mearsurment concentration(mg/mL)								Accuary(%)							
	Precision								Accuary(%)							
Nominal concentration (mg/mL)	Batch1	Batch2	Batch3	Batch4	Batch5	Mean	SD	CV	Batch1	Batch2	Batch3	Batch4	Batch5	Mean	SD	CV
0.0625	0.06	0.05	0.06	0.06	0.05	0.06	0.01	8.58	96.53	83.72	101.13	101.57	87.60	94.11	8.08	8.58
0.125	0.12	0.12	0.13	0.12	0.13	0.12	0.00	4.00	96.06	96.54	101.20	95.39	104.48	98.73	3.95	4.00
0.25	0.24	0.23	0.24	0.27	0.25	0.25	0.01	5.66	96.48	93.17	97.61	108.12	98.92	98.86	5.60	5.66
0.75	0.72	0.72	0.74	0.74	0.73	0.73	0.01	1.42	95.73	95.94	98.79	98.32	97.61	97.28	1.39	1.42

Table S15. The relationships of reaction times and OD values at each concentration

	0.059mM				0.119mM				0.238mM				0.342mM				0.684mM				0.950mM				1.900mM			
Time (h)	A6	A7	mean	SD	B6	B7	mean	SD	C6	C7	mean	SD	D6	D7	mean	SD	E6	E7	mean	SD	F6	F7	mean	SD	G6	G7	mean	SD
0.01	0.1930	0.1700	0.1815	0.0163	0.1830	0.1810	0.1820	0.0014	0.1800	0.2130	0.1965	0.0233	0.2160	0.1830	0.1995	0.0233	0.2110	0.1840	0.1975	0.0191	0.1930	0.1900	0.1915	0.0021	0.1850	0.1930	0.1890	0.0057
0.09	0.1740	0.1580	0.1660	0.0113	0.1710	0.1560	0.1635	0.0106	0.1510	0.1700	0.1605	0.0134	0.1770	0.1660	0.1715	0.0078	0.2070	0.1690	0.1880	0.0269	0.1770	0.1710	0.1740	0.0042	0.2460	0.1760	0.2110	0.0495
0.18	0.1540	0.1480	0.1510	0.0042	0.1520	0.1500	0.1510	0.0014	0.1520	0.1560	0.1540	0.0028	0.1620	0.1570	0.1595	0.0035	0.1660	0.1640	0.1650	0.0014	0.1680	0.1630	0.1655	0.0035	0.1830	0.1710	0.1770	0.0085
0.26	0.1550	0.1460	0.1505	0.0064	0.1510	0.1510	0.1510	0.0000	0.1530	0.1590	0.1560	0.0042	0.1660	0.1600	0.1630	0.0042	0.1700	0.1650	0.1675	0.0035	0.1760	0.1670	0.1715	0.0064	0.1790	0.1790	0.1790	0.0000
0.34	0.1560	0.1480	0.1520	0.0057	0.1520	0.1510	0.1515	0.0007	0.1550	0.1610	0.1580	0.0042	0.1690	0.1630	0.1660	0.0042	0.1750	0.1700	0.1725	0.0035	0.1820	0.1720	0.1770	0.0071	0.1860	0.1860	0.1860	0.0000
0.43	0.1560	0.1480	0.1520	0.0057	0.1550	0.1540	0.1545	0.0007	0.1580	0.1650	0.1615	0.0049	0.1740	0.1690	0.1715	0.0035	0.1820	0.1790	0.1805	0.0021	0.1910	0.1800	0.1855	0.0078	0.1940	0.1970	0.1955	0.0021
0.51	0.1580	0.1490	0.1535	0.0064	0.1570	0.1600	0.1585	0.0021	0.1630	0.1700	0.1665	0.0049	0.1800	0.1740	0.1770	0.0042	0.1900	0.1870	0.1885	0.0021	0.2000	0.1900	0.1950	0.0071	0.2060	0.2090	0.2075	0.0021
0.59	0.1580	0.1510	0.1545	0.0049	0.1590	0.1600	0.1595	0.0007	0.1680	0.1740	0.1710	0.0042	0.1870	0.1810	0.1840	0.0042	0.1990	0.1970	0.1980	0.0014	0.2080	0.2000	0.2040	0.0057	0.2190	0.2220	0.2205	0.0021
0.68	0.1600	0.1520	0.1560	0.0057	0.1610	0.1620	0.1615	0.0007	0.1730	0.1800	0.1765	0.0049	0.1940	0.1890	0.1915	0.0035	0.2090	0.2060	0.2075	0.0021	0.2200	0.2110	0.2155	0.0064	0.2320	0.2350	0.2335	0.0021
0.76	0.1610	0.1530	0.1570	0.0057	0.1640	0.1650	0.1645	0.0007	0.1770	0.1850	0.1810	0.0057	0.2020	0.1960	0.1990	0.0042	0.2200	0.2170	0.2185	0.0021	0.2310	0.2220	0.2265	0.0064	0.2460	0.2500	0.2480	0.0028
0.84	0.1630	0.1550	0.1590	0.0057	0.1670	0.1680	0.1675	0.0007	0.1830	0.1910	0.1870	0.0057	0.2110	0.2040	0.2075	0.0049	0.2310	0.2280	0.2295	0.0021	0.2450	0.2350	0.2400	0.0071	0.2610	0.2640	0.2625	0.0021
0.93	0.1640	0.1590	0.1615	0.0035	0.1700	0.1710	0.1705	0.0007	0.1880	0.1970	0.1925	0.0064	0.2190	0.2130	0.2160	0.0042	0.2420	0.2400	0.2410	0.0014	0.2580	0.2480	0.2530	0.0071	0.2780	0.2810	0.2795	0.0021
1.01	0.1650	0.1570	0.1610	0.0057	0.1730	0.1730	0.1730	0.0000	0.1930	0.2020	0.1975	0.0064	0.2280	0.2210	0.2245	0.0049	0.2550	0.2530	0.2540	0.0014	0.2700	0.2610	0.2655	0.0064	0.2940	0.2980	0.2960	0.0028
1.09	0.1670	0.1580	0.1625	0.0064	0.1750	0.1750	0.1750	0.0000	0.1990	0.2080	0.2035	0.0064	0.2370	0.2300	0.2335	0.0049	0.2670	0.2650	0.2660	0.0014	0.2840	0.2750	0.2795	0.0064	0.3110	0.3140	0.3125	0.0021
1.18	0.1680	0.1590	0.1635	0.0064	0.1780	0.1780	0.1780	0.0000	0.2050	0.2140	0.2095	0.0064	0.2460	0.2380	0.2420	0.0057	0.2790	0.2780	0.2785	0.0007	0.2980	0.2880	0.2930	0.0071	0.3280	0.3310	0.3295	0.0021
1.26	0.1680	0.1590	0.1635	0.0064	0.1810	0.1810	0.1810	0.0000	0.2100	0.2200	0.2150	0.0071	0.2550	0.2470	0.2510	0.0057	0.2920	0.2910	0.2915	0.0007	0.3120	0.3020	0.3070	0.0071	0.3440	0.3490	0.3465	0.0035
1.34	0.1690	0.1610	0.1650	0.0057	0.1830	0.1830	0.1830	0.0000	0.2160	0.2250	0.2205	0.0064	0.2630	0.2560	0.2595	0.0049	0.3040	0.3040	0.3040	0.0000	0.3270	0.3160	0.3215	0.0078	0.3620	0.3670	0.3645	0.0035
1.43	0.1700	0.1620	0.1660	0.0057	0.1850	0.1850	0.1850	0.0000	0.2200	0.2300	0.2250	0.0071	0.2720	0.2640	0.2680	0.0057	0.3180	0.3170	0.3175	0.0007	0.3410	0.3300	0.3355	0.0078	0.3800	0.3840	0.3820	0.0028
1.51	0.1700	0.1630	0.1665	0.0049	0.1870	0.1870	0.1870	0.0000	0.2260	0.2340	0.2300	0.0057	0.2800	0.2720	0.2760	0.0057	0.3290	0.3300	0.3295	0.0007	0.3550	0.3440	0.3495	0.0078	0.3970	0.4020	0.3995	0.0035
1.59	0.1710	0.1630	0.1670	0.0057	0.1890	0.1890	0.1890	0.0000	0.2300	0.2390	0.2345	0.0064	0.2880	0.2790	0.2835	0.0064	0.3420	0.3430	0.3425	0.0007	0.3690	0.3580	0.3635	0.0078	0.4140	0.4200	0.4170	0.0042
1.68	0.1720	0.1640	0.1680	0.0057	0.1910	0.1910	0.1910	0.0000	0.2340	0.2430	0.2385	0.0064	0.2960	0.2870	0.2915	0.0064	0.3550	0.3550	0.3550	0.0000	0.3840	0.3720	0.3780	0.0085	0.4330	0.4380	0.4355	0.0035
1.76	0.1730	0.1640	0.1685	0.0064	0.1930	0.1920	0.1925	0.0007	0.2390	0.2480	0.2435	0.0064	0.3030	0.2940	0.2985	0.0064	0.3670	0.3670	0.3670	0.0000	0.3990	0.3870	0.3930	0.0085	0.4510	0.4560	0.4535	0.0035
1.84	0.1730	0.1650	0.1690	0.0057	0.1940	0.1940	0.1940	0.0000	0.2420	0.2520	0.2470	0.0071	0.3110	0.3020	0.3065	0.0064	0.3790	0.3800	0.3795	0.0007	0.4130	0.4000	0.4065	0.0092	0.4670	0.4740	0.4705	0.0049
1.93	0.1730	0.1650	0.1690	0.0057	0.1960	0.1950	0.1955	0.0007	0.2450	0.2550	0.2500	0.0071	0.3180	0.3070	0.3125	0.0078	0.3910	0.3920	0.3915	0.0007	0.4260	0.4140	0.4200	0.0085	0.4860	0.4920	0.4890	0.0042
2.01	0.1730	0.1650	0.1690	0.0057	0.1970	0.1960	0.1965	0.0007	0.2490	0.2590	0.2540	0.0071	0.3250	0.3150	0.3200	0.0071	0.4030	0.4040	0.4035	0.0007	0.4410	0.4270	0.4340	0.0099	0.5020	0.5100	0.5060	0.0057
2.09	0.1740	0.1660	0.1700	0.0057	0.1980	0.2040	0.2010	0.0042	0.2530	0.2620	0.2575	0.0064	0.3310	0.3210	0.3260	0.0071	0.4140	0.4160	0.4150	0.0014	0.4550	0.4410	0.4480	0.0099	0.5210	0.5280	0.5245	0.0049
2.18	0.1740	0.1660	0.1700	0.0057	0.1990	0.1980	0.1985	0.0007	0.2550	0.2650	0.2600	0.0071	0.3370	0.3260	0.3315	0.0078	0.4250	0.4270	0.4260	0.0014	0.4670	0.4540	0.4605	0.0092	0.5370	0.5450	0.5410	0.0057
2.26	0.1740	0.1660	0.1700	0.0057	0.2000	0.1990	0.1995	0.0007	0.2570	0.2670	0.2620	0.0071	0.3430	0.3320	0.3375	0.0078	0.4370	0.4390	0.4380	0.0014	0.4800	0.4670	0.4735	0.0092	0.5540	0.5630	0.5585	0.0064
2.34	0.1740	0.1660	0.1700	0.0057	0.2010	0.2000	0.2005	0.0007	0.2600	0.2700	0.2650	0.0071	0.3490	0.3380	0.3435	0.0078	0.4470	0.4500	0.4485	0.0021	0.4940	0.4790	0.4865	0.0106	0.5710	0.5800	0.5755	0.0064
2.43	0.1760	0.1660	0.1710	0.0071	0.2020	0.2010	0.2015	0.0007	0.2630	0.2720	0.2675	0.0064	0.3540	0.3430	0.3485	0.0078	0.4580	0.4600	0.4590	0.0014	0.5070	0.4920	0.4995	0.0106	0.5880	0.5980	0.5930	0.0071
2.51	0.1740	0.1660	0.1700	0.0057	0.2020	0.2010	0.2015	0.0007	0.2650	0.2740	0.2695	0.0064	0.3580	0.3480	0.3530	0.0071	0.4680	0.4710	0.4695	0.0021	0.5190	0.5040	0.5115	0.0106	0.6050	0.6140	0.6095	0.0064
2.59	0.1740	0.1660	0.1700	0.0057	0.2030	0.2020	0.2025	0.0007	0.2670	0.2760	0.2715	0.0064	0.3630	0.3510	0.3570	0.0085	0.4780	0.4810	0.4795	0.0021	0.5320	0.5160	0.5240	0.0113	0.6220	0.6310	0.6265	0.0064
2.68	0.1740	0.1660	0.1700	0.0057	0.2030	0.2020	0.2025	0.0007	0.2690	0.2770	0.2730	0.0057	0.3680	0.3560	0.3620	0.0085	0.4870	0.4910	0.4890	0.0028	0.5440	0.5280	0.5360	0.0113	0.6390	0.6480	0.6435	0.0064
2.76	0.1740	0.1660	0.1700	0.0057	0.2040	0.2030	0.2035	0.0007	0.2700	0.2790	0.2745	0.0064	0.3710	0.3600	0.3655	0.0078	0.4970	0.5000	0.4985	0.0021	0.5560	0.5390	0.5475	0.0120	0.6550	0.6640	0.6595	0.0064
2.84	0.1740	0.1660	0.1700	0.0057	0.2040	0.2030	0.2035	0.0007	0.2720	0.2800	0.2760	0.0057	0.3750	0.3640	0.3695	0.0078	0.5060	0.5090	0.5075	0.0021	0.5680	0.5500	0.5590	0.0127	0.6710	0.6800	0.6755	0.0064
2.93	0.1740	0.1660	0.1700	0.0057	0.2040	0.2030	0.2035	0.0007	0.2730	0.2820	0.2775	0.0064	0.3780	0.3660	0.3720	0.0085	0.5150	0.5180	0.5165	0.0021	0.5790	0.5610	0.5700	0.0127	0.6860	0.6970	0.6915	0.0078
3.01	0.1740	0.1660	0.1700	0.0057	0.2050	0.2030	0.2040	0.0014	0.2740	0.2830	0.2785	0.0064	0.3820	0.3700	0.3760	0.0085	0.5220	0.5270	0.5245	0.0035	0.5900	0.5720	0.5810	0.0127	0.7010	0.7120	0.7065	0.0078
3.09	0.1730	0.1650	0.1690	0.0057	0.2050	0.2030	0.2040	0.0014	0.2750	0.2850	0.2800	0.0071	0.3850	0.3720	0.3785	0.0092	0.5310	0.5350	0.5330	0.0028	0.6010	0.5820	0.5915	0.0134	0.7170	0.7280	0.7225	0.0078
3.18	0.1730	0.1650	0.1690	0.0057	0.2050	0.2040	0.2045	0.0007	0.2760	0.2850	0.2805	0.0064	0.3880	0.3740	0.3810	0.0099	0.5380	0.5430	0.5405	0.0035	0.6110	0.5920	0.6015	0.0134	0.7330	0.7430	0.7380	0.0071
3.26	0.1730	0.1650	0.1690	0.0057	0.2050	0.2040	0.2045	0.0007	0.2770	0.2850	0.2810	0.0057	0.3900	0.3770	0.3835	0.0092	0.5460	0.5500	0.5480	0.0028	0.6210	0.6010	0.6110	0.0141	0.7470	0.7570	0.7520	0.0071
3.34	0.1730	0.1650	0.1690	0.0057	0.2050	0.2040	0.2045	0.0007	0.2780	0.2860	0.2820	0.0057	0.3920	0.3800	0.3860	0.0085	0.5530	0.5570	0.5550	0.0028	0.6300	0.6100	0.6200	0.0141	0.7620	0.7720	0.7670	0.0071
3.43	0.1720	0.1640	0.1680	0.0057	0.2050	0.2040	0.2045	0.0007	0.2780	0.2870	0.2825	0.0064	0.3950	0.3810	0.3880	0.0099	0.5590	0.5640	0.5615	0.0035	0.6400	0.6190	0.6295	0.0148	0.7770	0.7870	0.7820	0.0071
3.51	0.1720	0.1640	0.1680	0.0057	0.2050	0.2040	0.2045	0.0007	0.2790	0.2870	0.2830	0.0057	0.3970	0.3820	0.3895	0.0106	0.5650	0.5700	0.5675	0.0035	0.6490	0.6280	0.6385	0.0148	0.7900	0.8010	0.7955	0.0078
3.59	0.1720	0.1640	0.1680	0.0057	0.2050	0.2030	0.2040	0.0014	0.2790	0.2870	0.2830	0.0057	0.3970	0.3840	0.3905	0.0092	0.5710	0.5760	0.5735	0.0035	0.6580	0.6360	0.6470	0.0156	0.8030	0.8150	0.8090	0.0085
3.68	0.1720	0.1630	0.1675	0.0064	0.2050	0.2030	0.2040	0.0014	0.2790	0.2880	0.2835	0.0064	0.3990	0.3850	0.3920	0.0099	0.5770	0.5820	0.5795	0.0035	0.6670	0.6440	0.6555	0.0163	0.8180	0.8290	0.8235	0.0078
3.76	0.1710	0.1630	0.1670	0.0057	0.2050	0.2030	0.2040	0.0014	0.2800	0.2880	0.2840	0.0057	0.4000	0.3860	0.3930	0.0099	0.5820	0.5870	0.5845	0.0035	0.6740	0.6510	0.6625	0.0163	0.8290	0.8420	0.8355	0.0092
3.84	0.1720	0.1630	0.1675	0.0064	0.2050	0.2030	0.2040	0.0014	0.2800	0.2880	0.2840	0.0057	0.4010	0.3870	0.3940	0.0099	0.5870	0.5920	0.5895	0.0035	0.6810	0.6580	0.6695	0.0163	0.8420	0.8540	0.8480	0.0085
3.93	0.1710	0.1620	0.1665	0.0064	0.2050	0.2030	0.2040	0.0014	0.2800	0.2880	0.2840	0.0057	0.4020	0.3880	0.3950	0.0099	0.5910	0.5960	0.5935	0.0035	0.6890	0.6650	0.6770	0.0170	0.8540	0.8670	0.8605	0.0092
4.01	0.1700	0.1620	0.1660	0.0057	0.2040	0.2030	0.2035	0.0007	0.2810	0.2880	0.2845	0.0049	0.4030	0.3890	0.3960	0.0099	0.5950	0.6010	0.5980	0.0042	0.6950	0.6710	0.6830	0.0170	0.8670	0.8790	0.8730	0.0085

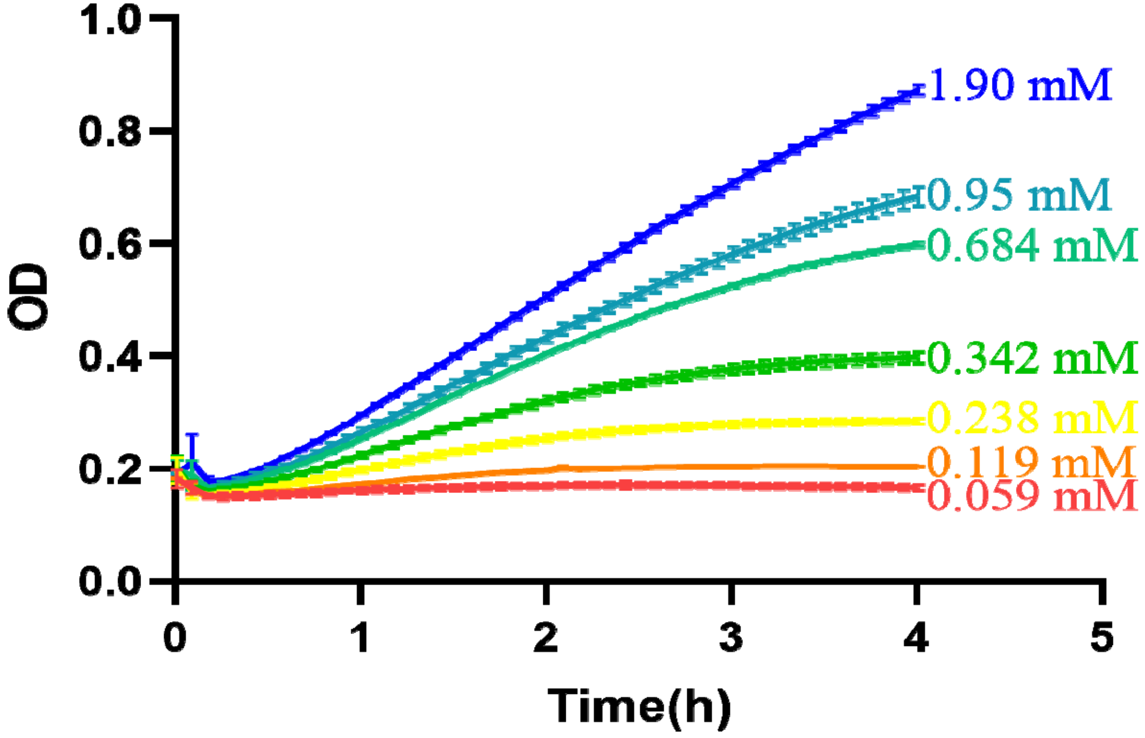

Fig.S2 The time-OD value relationship curves at each concentration

Table S16. OD values at different temperatures and different pH

pH	25℃	30℃	35℃	37℃	40℃
7.00	0.007500	0.009000	0.013417	0.016833	0.017500
7.40	0.006333	0.009667	0.015000	0.017667	0.019917
8.00	0.005917	0.010167	0.016167	0.017917	0.021583
8.50	0.006167	0.011167	0.017333	0.018833	0.022083
8.70	0.006167	0.011083	0.017417	0.019250	0.021917
9.00	0.006917	0.011583	0.016917	0.018750	0.021583

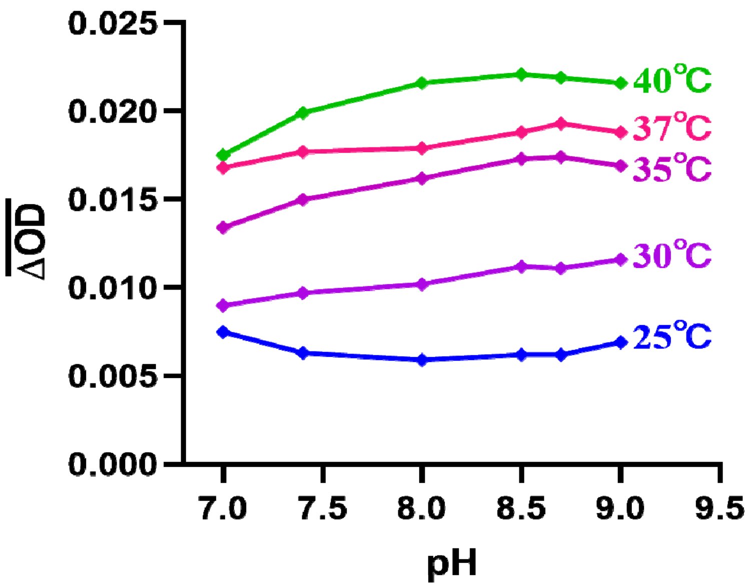

Fig. S3 The pH- $\Delta\overline{OD}$ value relationship curves at different temperatures

Table S17. The original data of 5-OH SAX groups and control groups: DPP-4 inhibition ratio(a), Glucose(b) and Insulin(c)

Fig. S4 A scatter plot of 5-OH SAX groups and control groups: DPP-4 inhibition ratio(a), Glucose(b) and Insulin(c)

Table S17-a. DPP-4 inhibition ratio

Time(h)	Subject1	Subject2	Subject3	Control1	Control2	Control3	P
0.00	0	0	0	0	0	0	
0.03	68.62%		69.58%	5.56%	6.25%		0.00
0.10	62.41%	56.23%	67.83%		-14.58%	-2.65%	0.00
0.16	56.90%	55.09%	58.74%	5.56%	-13.89%	-3.31%	0.00
0.25	52.41%	44.91%	55.59%	3.17%	-3.47%	-1.99%	0.00
0.50	44.83%	26.42%	33.92%	1.59%	-20.83%	-10.60%	0.01
0.83	27.59%	19.25%	32.52%	7.14%	-6.25%	3.31%	0.01
1.05	27.93%	13.58%	26.92%	18.25%	-6.94%	9.93%	0.15
1.50	10.69%	13.96%	22.73%	9.52%	2.78%	11.26%	0.15
2.00	3.45%	3.40%	9.79%	6.35%	-4.86%	9.27%	0.70
3.00	-3.45%	0.38%	33.22%	8.73%	-6.25%	3.97%	0.56
4.00	-7.24%	17.74%	19.93%	0.79%	2.08%	-1.99%	0.33
6.00	-13.79%	4.91%	15.38%	7.94%	-8.33%	6.62%	0.99
8.00	1.38%	17.36%	19.58%	8.73%	4.86%	7.28%	0.38
10.00	-7.59%	1.13%	14.69%	19.84%	-0.69%	12.58%	0.43
12.00	22.07%	14.72%	24.48%	34.92%	17.36%	11.92%	0.90
24.00	-8.97%	12.08%	35.31%	15.87%	11.11%	0.00%	0.79

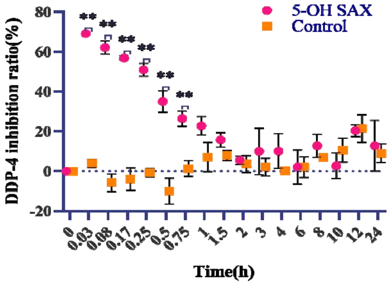

Fig. S4-a DPP-4 inhibition ratio

Table S17-b. Glucose

Time(h)	Subject1	Subject2	Subject3	Control1	Control2	Control3	P
0.00	10.5683	6.8096	8.5164	7.8421	38.4903	33.7493	0.1326
0.03	13.7919		12.1287	16.8303	29.8056	31.1857	0.1166
0.10	15.6862	9.2247	9.7843	17.9892	33.6597	25.0279	0.0483
0.16	17.0931	7.2548	8.4685	16.7788	29.2146	30.1021	0.0529
0.25	14.7557	8.8933	11.9373	19.4057	33.0944	32.2164	0.0259
0.50	15.7749	7.6526	13.3009	17.9892	32.2722	29.8907	0.0451
0.83	14.9772	7.8799	15.9084	17.2939	32.3750	31.7143	0.0622
1.05	16.1626	6.0993	17.4873	16.3925	38.0792	29.0186	0.1144
1.50	19.5746	5.2564	11.5784	15.1563	37.0771	32.6657	0.1093
2.00	19.9180	7.0275	19.9513	15.2078	32.0924	35.1764	0.1914
3.00	18.9653	7.5294	14.7123	14.1003	30.8333	30.9214	0.1508
4.00	18.0680	7.4063	17.6308	17.6029	28.9833	36.2600	0.1092
6.00	17.9018	7.8325	15.4060	14.7442	28.8806	35.1764	0.1368
8.00	14.8997	7.5768	18.5877	10.5463	31.3215	29.2829	0.2447
10.00	8.3859	7.8799	14.7601	7.8421	31.6813	30.0229	0.1837
12.00	7.1784	8.2113	13.6358	8.8723	29.2146	17.3900	0.2298
24.00	21.0036	10.7590	7.8226	20.4358	37.6938	42.9464	0.0601

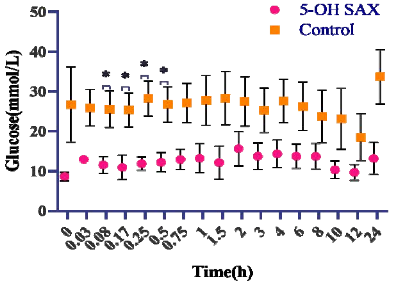

Fig. S4-b Glucose

Table S17-c. Insulin

Time(h)	Subject1	Subject2	Subject3	Control1	Control2	Control3	P
0.00	24.1045	10.0747	16.3223	18.5878	24.2754	28.5354	0.2342
0.03	19.8756	4.4425	14.4436	14.3986	24.7101	23.9714	0.2219
0.10	31.4013	11.5115	15.9610	20.5473	20.3623	21.0422	0.8732
0.16	20.0415	13.6379	13.9379	26.4932	17.4638	23.6308	0.1205
0.25	25.1824		11.1199	20.2770	18.5507	19.8161	0.8086
0.50	19.4610	11.9138	12.2038	19.8041	15.5072	19.2030	0.2644
0.83	31.3184	7.5460	18.3454	20.8851	21.8116	43.2493	0.3936
1.05	20.2073	9.9023	12.5650	24.0608	19.0580	35.8924	0.1080
1.50	27.0066	8.4655	17.1171	19.1284	19.6377	25.2657	0.5404
2.00	26.1774	8.1207	12.7095	24.1284	20.9420	18.5899	0.3816
3.00	26.3433	15.0747	13.5766	21.2905	16.2319	36.9142	0.4315
4.00	23.2753	10.5920	12.5650	23.3176	21.2319	22.7452	0.1562
6.00	24.0216	6.7989	14.6604	24.8041	22.4638	21.0422	0.2098
8.00	16.4760	5.1897	12.7095	15.1419	21.0145	17.7725	0.1552
10.00	24.2703	4.7874	11.9870	16.4257	18.7681	18.8624	0.4922
12.00	22.6949	8.0057	16.1777	21.5608	18.1884	19.8842	0.3846
24.00	20.2073	10.5920	12.7818	21.4257	19.0580	17.2275	0.2095

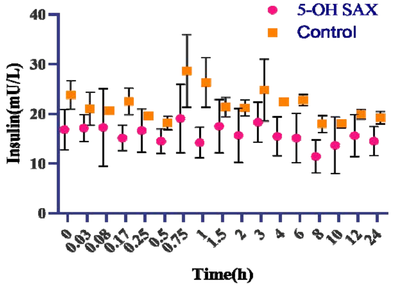

Fig. S4-c Insulin

Table S18. The original data of SAX groups and control groups: DPP-4 inhibition ratio(a), Glucose(b) and Insulin(c)

Table S18-a. DPP-4 inhibition ratio

Time(h)	Subject1	Subject2	Subject3	Control1	Control2	Control3	P
0.00	0	0	0	0	0	0	
0.08	68.12%	71.91%	76.12%	1.75%	1.87%	-20.16%	0.00
0.17	62.90%	72.88%	66.57%	6.43%	5.62%	-28.23%	0.00
0.25	66.67%	72.64%	66.29%	0.58%	10.62%	-0.81%	0.00
0.50	68.41%	69.73%	58.15%	-0.58%	5.62%	-18.55%	0.00
0.75	59.13%	63.20%	55.34%	8.77%	10.00%	-16.13%	0.00
1.00	61.45%	67.31%	54.78%	13.45%	15.63%	-13.71%	0.01
1.50	65.22%	69.01%	56.46%	19.30%	33.12%	-8.06%	0.02
2.00	65.51%	58.84%	64.04%	18.13%	23.13%	0.81%	0.00
3.00	61.74%	62.23%	58.71%	12.28%	17.50%	-1.61%	0.00
4.00	53.91%	55.69%	54.49%	18.71%	9.37%	-6.45%	0.00
6.00	45.51%	46.00%	50.00%	18.71%	12.50%	1.61%	0.00
8.00	33.33%	68.04%	20.51%	14.04%	15.63%	-11.29%	0.11
10.00	31.88%	49.39%	28.09%	22.81%	18.75%	-6.45%	0.09
12.00	38.84%	55.93%	33.71%	25.15%	11.25%	1.61%	0.03
24.00	34.20%	32.45%	12.08%	19.88%	-10.62%	5.65%	0.13

Table S18-b. Glucose

Time(h)	Subject1	Subject2	Subject3	Control1	Control2	Control3	P
0.00	14.3244	3.1471	29.1056	28.3710	8.3055	26.3722	0.6073
0.08		3.4719	33.4179	25.3178	12.9847	28.8418	0.7780
0.17	8.0295	2.9611	43.3588	30.9066	13.2706	38.4861	0.5568
0.25	6.9959	5.2000	35.7852	22.2906	14.9863	38.2521	0.4887
0.50	6.5286	8.1421	34.0073	25.9906	13.0626	32.0132	0.5170
0.75	8.7579	8.7066	42.8873	24.6710	15.4542	35.8345	0.7056
1.00	8.0735	6.1145	42.4845	21.3332	14.5444	27.5160	0.8653
1.50	8.3374	6.1901	39.5769	23.0150	14.7523	26.8922	0.7724
2.00	8.3676	5.2315	31.3943	19.1339	14.6224	32.7931	0.5079
3.00	5.8331	5.9757	37.6614	21.2038	14.4404	24.5006	0.7624
4.00	5.5912	6.7325	23.8699	23.6360	19.7434	26.9181	0.1438
6.00	7.1801	3.9386	30.9818	27.1031	23.3568	31.3114	0.2086
8.00	9.4122	4.7837	38.3883	27.2584	22.9149	26.2683	0.4947
10.00	10.5447	2.8255	35.2646	18.7717	29.8816	33.1310	0.3603
12.00	6.1493	7.9340	57.8379	18.3318	16.3641	26.0083	0.8385
24.00	5.6490	10.6112	41.6790	39.1346	34.7687	33.9369	0.2179

Table S18-c. Insulin

Time(h)	Subject1	Subject2	Subject3	Control1	Control2	Control3	P
0.00	20.6731	12.7250	20.2912	18.7599	23.9076	13.0572	0.8755
0.08	22.1591	24.5090	22.9227	21.3984	20.9018	13.7170	0.1539
0.17	19.2745	22.5450	15.3080	21.3325	21.1950	19.3622	0.5081
0.25	17.4388	21.3175	19.0594	23.1794	21.1217	15.6965	0.7855
0.50		18.9444	17.1557	17.5066	15.1833	18.8490	0.6103
0.75	25.8304	18.5352	12.4524	19.6174	12.6173	17.6760	0.6280
1.00	33.0857	20.0082	16.7637	14.6702	21.2683	16.8695	0.3482
1.50	28.0157	18.6989	27.6260	21.0026	21.3416	13.6437	0.1959
2.00	28.3654	21.0720	20.6831	19.8153	19.2155	19.1422	0.1874
3.00		17.3077	17.0437	18.7599	20.3152	16.5029	0.4153
4.00		12.5614	19.1153	16.9789	16.9428	20.0220	0.4963
6.00	25.1311	15.2619	19.8992	19.3536	25.5938	16.4296	0.9311
8.00	22.6836	18.3715	17.5476	14.2744	17.8959	15.4765	0.1293
10.00	24.2570	18.2079	15.8119	16.5172	19.1422	9.6114	0.3169
12.00	39.4668	16.4075	12.3964	20.9367	12.4707	20.3886	0.6153
24.00	19.2745	15.0164	13.8522	20.1451	20.2419	18.6290	0.1041

Fig. S5 A scatter plot of SAX groups and control groups: DPP-4 inhibition ratio(a), Glucose(b) and Insulin(c)

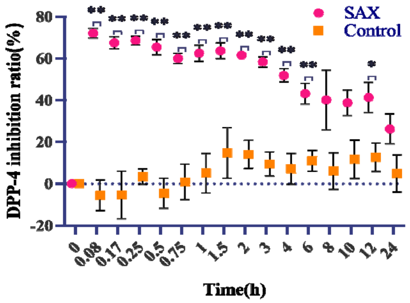

Fig. S5-a DPP-4 inhibition ratio

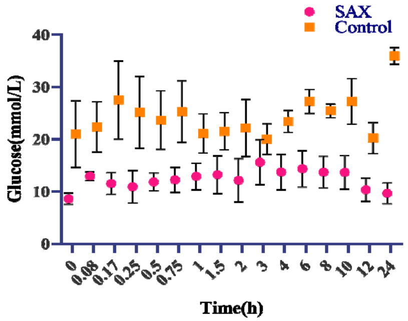

Fig. S5-b Glucose

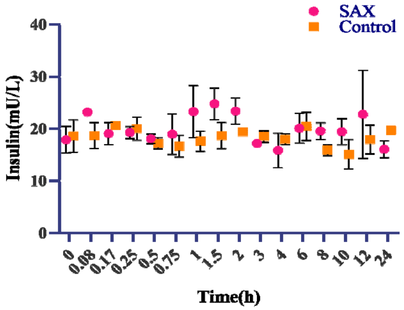

Fig. S5-c Insulin

Table S19. The data of PK/PD link model of 5-OH SAX in T2DM rats: Observed concentrations and inhibition ratios(a) and Parameter summary(b)

Table S19-a. Observed concentrations and inhibition ratios

	Subject1		Subject2		Subject3	
Nominal time(h)	C_{5ohsax}(ng/mL)	Inhibition ratio(%)	C_{5ohsax}(ng/mL)	Inhibition ratio(%)	C_{5ohsax}(ng/mL)	Inhibition ratio(%)
0.03	3400.00	68.62	2320.00		0.03	2850.00
0.08	1610.00	62.41	1240.00	56.23	0.10	1640.00
0.17	972.00	56.90	684.00	55.09	0.16	900.00
0.25	595.00	52.41	387.00	44.91	0.25	550.00
0.50	295.00	44.83	192.00	26.42	0.50	235.00
0.75	150.00	27.59	91.70	19.25	0.83	149.00
1.00	104.00		55.30	13.58	1.05	85.90
1.50	41.80	10.69	18.40		1.50	27.80
2.00	20.60	3.45	8.16	3.40	2.00	11.60

Table S19-b.Parameter summary

Parameter	Subject1	Subject2	Subject3	Mean	SD	CV
C_{max}	4902.67	2855.70	3392.28	3716.88	1061.39	28.56
AUC (ng*h/mL)	691.49	452.17	611.48	585.04	121.83	20.82
$t_{1/2a}$(h)	0.05	0.06	0.07	0.06	0.01	16.50
$t_{1/2\beta}$(h)	0.39	0.33	0.37	0.36	0.03	7.88
V(ml/kg)	101.99	175.09	147.39	141.49	36.91	26.09
V_2(ml/kg)	145.22	145.45	122.77	137.81	13.03	9.46
Cl(ml/h/kg)	723.08	1105.79	817.70	882.19	199.34	22.60
Cl_2(ml/h/kg)	494.78	513.24	406.78	471.60	56.89	12.06
K_{12}(1/h)	4.85	2.93	2.76	3.51	1.16	33.05
K_{21}(1/h)	3.41	3.53	3.31	3.42	0.11	3.16
EC_{50}(ng/mL)	187.15	204.41	363.66	251.74	97.31	38.65
Gam	1.01	1.42	1.50	1.31	0.26	20.10
E_0	-3.85	3.78	21.15	7.03	12.81	182.30
E_{max}	74.71	57.67	50.25	60.88	12.54	20.60
stdev0	1.84	1.94	2.14	1.97	0.15	7.79

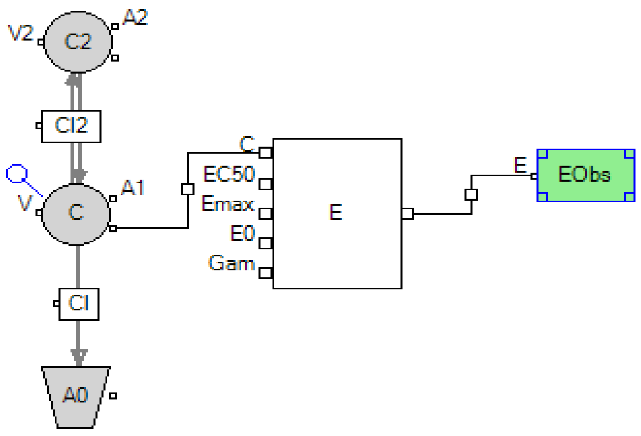

Fig. S6 Schematic representation of the PK/PD model of 5-OH SAX in T2DM rats

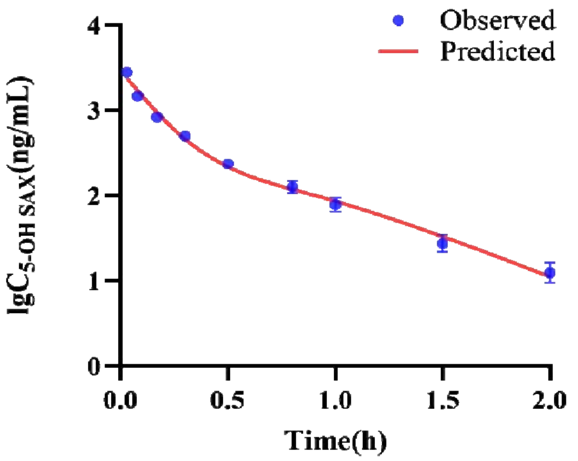

Fig.S7 The PK profiles of 5-OH SAX in T2DM rats

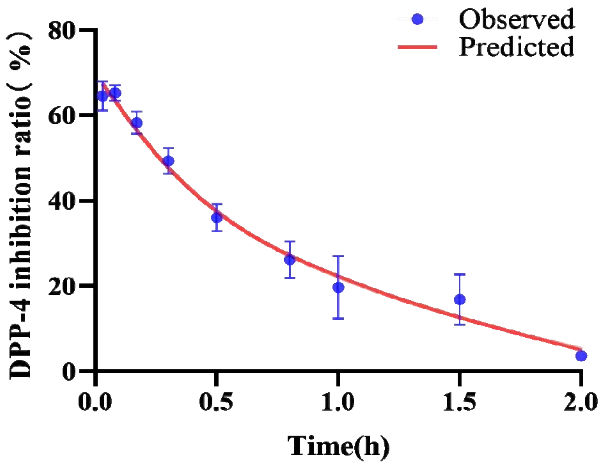

Fig.S8 The PD profiles of 5-OH SAX in T2DM rats

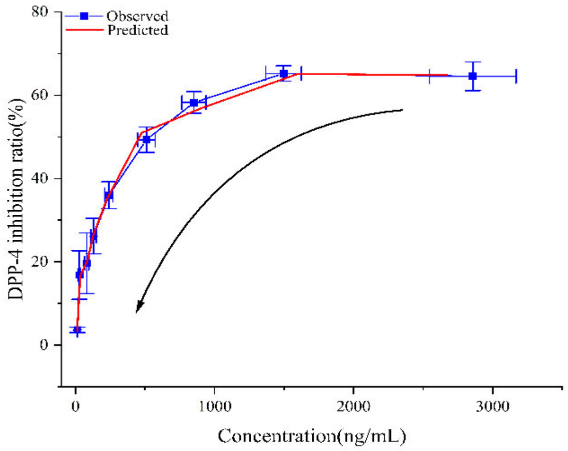

Fig.S9 The dose-effect curve of 5-OH SAX in T2DM rats

Table S20. Observed concentrations vs. Predicted concentrations of 5-OH SAX in T2DM rats

	Observed concentration (ng/mL)						Lg (Observed concentration)			Predicted concentration (ng/mL)						Lg (Predicted concentration)		
Nominal time(h)	1	2	3	Mean	SD	CV	Mean	SD	CV	1	2	3	Mean	SD	CV	Mean	SD	CV
0.03	3400.0	2320.0	2850.0	2856.7	540.03	311.79	3.45	0.08	0.05	3326.14	2111.09	2585.57	2674.27	612.36	353.55	3.42	0.10	0.06
0.08	1610.0	1240.0	1640.0	1496.7	222.79	128.63	3.17	0.07	0.04	1654.35	1377.80	1760.15	1597.43	197.43	113.98	3.20	0.06	0.03
0.17	972.0	684.0	900.0	852.0	149.88	86.53	2.93	0.08	0.05	982.73	738.58	998.04	906.45	145.58	84.05	2.95	0.07	0.04
0.30	595.0	387.0	550.0	510.7	109.44	63.18	2.70	0.10	0.06	575.69	352.56	499.41	475.89	113.41	65.48	2.67	0.11	0.06
0.50	295.0	192.0	235.0	240.7	51.73	29.87	2.37	0.09	0.05	282.57	180.32	214.24	225.71	52.08	30.07	2.35	0.10	0.06
0.80	150.0	91.7	149.0	130.2	33.37	19.27	2.10	0.12	0.07	153.51	90.88	141.68	128.69	33.27	19.21	2.10	0.12	0.07
1.00	104.0	55.3	85.9	81.7	24.62	14.21	1.90	0.14	0.08	104.34	59.65	93.36	85.78	23.29	13.45	1.92	0.13	0.07
1.50	41.8	18.4	27.8	29.3	11.78	6.80	1.44	0.18	0.10	46.83	21.02	36.25	34.70	12.97	7.49	1.52	0.18	0.10
2.00	20.6	8.2	11.6	13.5	6.42	3.71	1.10	0.20	0.12	19.23	7.41	10.29	12.31	6.16	3.56	1.06	0.21	0.12

Table S21. Observed inhibition ratios vs. Predicted inhibition ratios of 5-OH SAX in T2DM rats

	Observed inhibition ratio (%)						Predicted inhibition ratio (%)					
Nominal time(h)	1	2	3	Mean	SD	CV	1	2	3	Mean	SD	CV
0.03	68.62	56.23	69.58	64.54	5.90	3.40	66.95	57.82	68.86	64.81	7.45	4.30
0.08	62.41		67.83	65.21	2.61	1.85	63.36		67.06	65.12	3.83	2.71
0.17	56.90	55.09	58.74	58.24	4.50	2.60	59.01	53.40	62.31	56.91	1.82	1.05
0.30	52.41	44.91	55.59	49.32	5.29	3.06	52.63	43.22	52.12	50.97	5.49	3.17
0.50	44.83	26.42	33.92	36.01	5.58	3.22	41.14	30.07	36.82	35.05	9.26	5.35
0.80	27.59	19.25	32.52	26.16	7.37	4.25	29.79	17.68	31.01	26.45	6.71	3.87
1.00		13.58	26.92	19.67	10.32	7.30		12.37	26.96	20.25	9.43	6.67
1.50	10.69		22.73	16.85	8.27	5.85	11.00		22.70	16.71	8.51	6.02
2.00	3.45	3.40		3.66	0.91	0.64	3.02	4.31		3.42	0.04	0.03

Table S22. The data of PK/PD link model of SAX in T2DM rats:
Observed concentrations, total inhibition ratios and parent sax inhibition ratios(a) and Parameter summary(b)

Table S22-a. Observed concentrations, total inhibition ratios and parent sax inhibition ratios

	Subject1				Subject2				Subject3			
Nominal time(h)	C_{sax}(ng/mL)	C_{5ohsax}(ng/mL)	Total inhibition ratio(%)	Parent SAX inhibition ratio(%)	C_{sax}(ng/mL)	C_{5ohsax}(ng/mL)	Total inhibition ratio(%)	Parent SAX inhibition ratio(%)	C_{sax}(ng/mL)	C_{5ohsax}(ng/mL)	Total inhibition ratio(%)	Parent SAX inhibition ratio(%)
0.00	0.00	0.00	0.00	0.00	0.00	0.00	0.00	0.00	0.00	0.00	0.00	0.00
0.08	1550.00	6.22	68.12	60.61	3000.00	9.96	71.91	64.00	1420.00	24.30	76.12	
0.17	1260.00	47.20	62.90	49.72					1260.00	79.00	66.57	48.56
0.25	915.00	77.70	38.55	48.85	3260.00	46.50	72.64	59.57	609.00	115.00	66.29	43.17
0.50	422.00	166.00	68.41	39.02	1100.00	81.00	69.73	51.42	375.00	160.00	58.15	29.43
0.75	405.00	236.00	61.45	25.27	892.00	97.20	68.13	47.47	204.00	160.00	55.34	26.62
1.00	326.00	272.00	65.22	26.22	609.00	101.00	67.31	46.11	181.00	195.00	54.78	22.34
1.50	315.00	285.00	65.51	25.58	369.00	91.80	61.80	41.91	214.00	209.00	56.46	22.68
2.00	323.00	289.00	61.74	21.54	163.00	86.90	58.84	39.66			64.04	
3.00	314.00	318.00	53.91	11.84	171.00	163.00	62.23	33.18	266.00	334.00	50.59	21.31
4.00	213.00	210.00	45.51	11.63					189.00	235.00	54.49	18.40
6.00	90.10	92.40	24.68	4.70					152.00	165.00	50.00	20.72

Table S22-b. Parameter summary

Parameter	Subject1	Subject2	Subject3	Mean	SD	CV
C_{max} （ng/mL）	1549.63	3587.55	1454.21	2197.13	1205.08	54.85
T_{max}(h)	0.09	0.15	0.10	0.11	0.03	27.63
AUC (ng*h/mL)	2633.33	2677.30	4535.55	3282.06	1085.78	33.08
$t_{1/2Ka}$(h)	0.04	0.09	0.06	0.07	0.03	37.50
$t_{1/2a}$(h)	0.07	0.09	0.06	0.08	0.02	20.79
$t_{1/2\beta}$(h)	3.82	1.42	13.16	6.13	6.20	101.21
V(mL/kg)	3200.00	1081.89	2639.83	2307.24	1097.52	47.57
V_2(mL/kg)	15531.70	3457.89	36190.30	18393.30	16552.77	89.99

Cl(mL/h/kg)	3797.47	3735.11	2204.81	3245.80	902.06	27.79						
Cl_2(mL/h/kg)	22989.30	3587.65	24694.30	17090.42	11724.77	68.60						
K_{12}(1/h)	7.18	3.32	9.35	6.62	3.06	46.22						
K_{21}(1/h)	1.48	1.04	0.68	1.07	0.40	37.47						
EC_{50}	376.42	973.64	284.15	544.74	374.29	68.71						
Gam	2.52	0.39	1.23	1.38	1.08	77.98						
E_{max}	56.77	100.58	57.07	71.47	25.21	35.27						
stdev0	3.09	1.65	1.29	2.01	0.95	47.57						

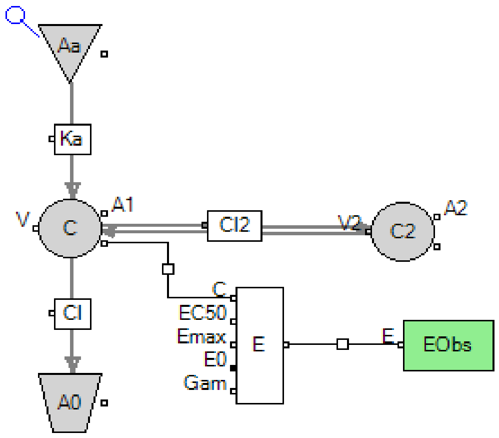

Fig. S10 Schematic representation of the PK/PD model of SAX in T2DM rats

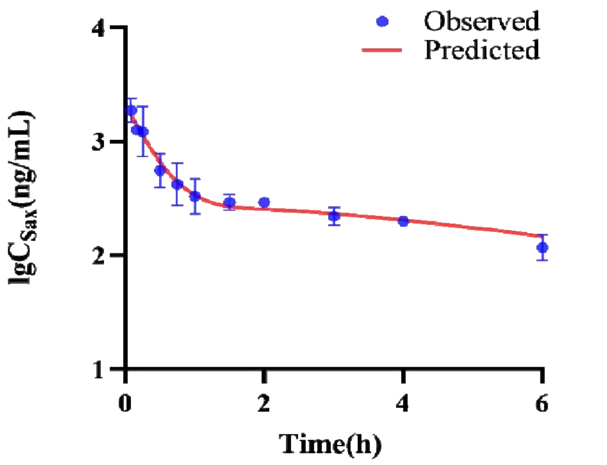

Fig.S11 The PK profiles of SAX in T2DM rats

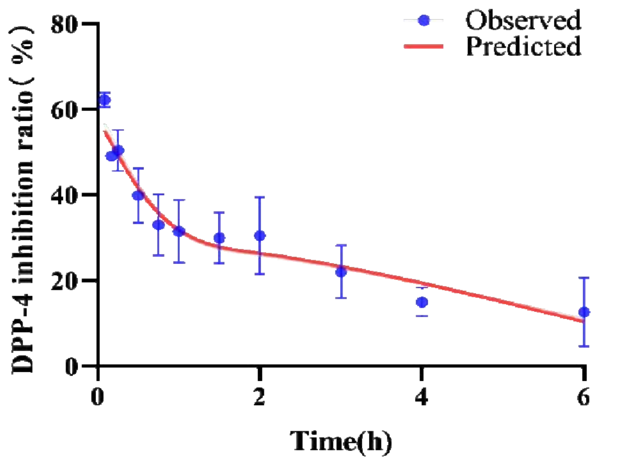

Fig.S12 The PD profiles of SAX in T2DM rats

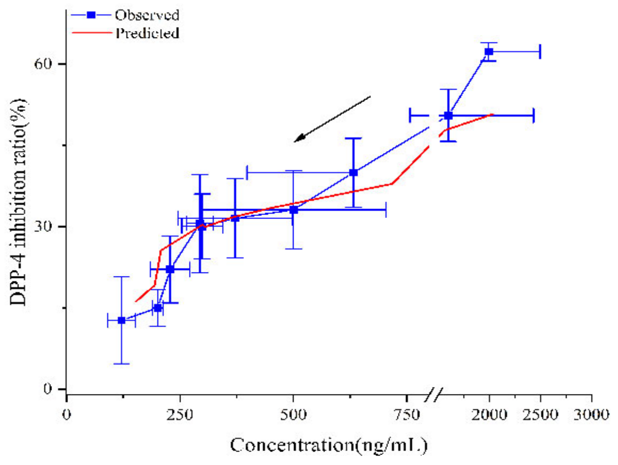

Fig.S13 The dose-effect curve of parent SAX in T2DM rats

Table S23. Observed concentrations vs. Predicted concentrations of SAX in T2DM rats

	Observed concentration (ng/mL)						Lg (Observed concentration)			Predicted concentration (ng/mL)						Lg (Predicted concentration)		
Nominal time(h)	1	2	3	Mean	SD	CV	Mean	SD	CV	1	2	3	Mean	SD	CV	Mean	SD	CV
0.08	1550.0	3000.0	1420.0	1990.00	877.10	506.39	3.27	0.18	0.10	1545.49	3092.90	1453.96	2030.78	920.96	531.72	3.28	0.18	0.11
0.17	1260.0		1260.0	1260.00	0.00	0.00	3.10	0.00	0.00	1276.71		1163.17	1219.94	80.28	56.77	3.09	0.03	0.02
0.25	915.0	3260.0	609.0	1594.67	1450.31	837.34	3.09	0.38	0.22	893.34	3072.42	718.76	1561.51	1311.40	757.14	3.10	0.34	0.20
0.50	422.0	1100.0	375.0	632.33	405.69	234.23	2.75	0.26	0.15	448.35	1403.66	302.13	718.05	598.24	345.40	2.76	0.35	0.20
0.75	405.0	892.0	204.0	500.33	353.77	204.25	2.62	0.32	0.19	382.56	740.54	222.59	448.56	265.21	153.12	2.60	0.26	0.15
1.00	326.0	609.0	181.0	372.00	217.68	125.68	2.52	0.26	0.15	361.29	528.11	209.93	366.44	159.15	91.89	2.53	0.20	0.12
1.50	315.0	369.0	214.0	299.33	78.68	45.43	2.47	0.12	0.07	329.51	383.08	205.54	306.04	91.07	52.58	2.47	0.14	0.08
2.00	323.0	265.1		294.04	40.96	28.96	2.47	0.06	0.04	299.99	298.72		299.35	0.90	0.64	2.48	0.00	0.00
3.00	314.0	171.0	201.1	228.69	75.40	43.53	2.34	0.14	0.08	250.92	183.05	189.92	207.96	37.36	21.57	2.31	0.07	0.04
4.00	213.0		189.0	201.00	16.97	12.00	2.30	0.04	0.03	208.60		180.17	194.39	20.10	14.22	2.29	0.04	0.03
6.00	90.1		152.0	121.05	43.77	30.95	2.07	0.16	0.11	144.62		162.02	153.32	12.30	8.70	2.18	0.03	0.02

Table S24. Observed inhibition ratios vs. Predicted inhibition ratios of SAX in T2DM rats

	Observed inhibition ratio（%）						Predicted inhibition ratio (%)					
Nominal time(h)	1	2	3	Mean	SD	CV	1	2	3	Mean	SD	CV
0.08	60.61	64.00		62.30	2.40	1.70	55.21	61.34	50.32	55.63	5.52	3.19
0.17	49.72		48.56	49.14	0.82	0.58	54.29		48.51	51.40	4.08	2.89
0.25	48.85	59.57	43.17	50.53	8.33	4.81	51.02	61.28	43.27	51.85	9.04	5.22
0.50	39.02	51.42	29.43	39.96	11.03	6.37	34.55	53.84	29.61	39.33	12.80	7.39
0.75	25.27	47.47	26.62	33.12	12.45	7.19	28.97	47.63	24.28	33.62	12.35	7.13
1.00	26.22	46.11	22.34	31.56	12.76	7.36	26.92	44.37	23.28	31.52	11.27	6.51
1.50	25.58	41.91	22.68	30.06	10.37	5.99	23.66	41.32	22.92	29.30	10.41	6.01
2.00	21.54	39.66		30.60	12.81	9.06	20.47	39.00		29.73	13.10	9.26
3.00	11.84	33.18	21.31	22.11	10.69	6.17	15.00	34.58	21.60	23.73	9.96	5.75
4.00	11.63		18.40	15.01	4.79	3.38	10.44		20.73	15.59	7.28	5.15
6.00	4.70		20.72	12.71	11.33	8.01	4.66		19.04	11.85	10.17	7.19

Table S25. The comparison of observed total DPP-4 inhibition ratios and parent SAX DPP-4 inhibition ratios after intragastric administration of 10 mg/kg SAX in T2DM rats

	Subject1		Subject2		Subject3	
Nominal time(h)	Total inhibition ratio(%)	Parent SAX inhibition ratio(%)	Total inhibition ratio(%)	Parent SAX inhibition ratio(%)	Total inhibition ratio(%)	Parent SAX inhibition ratio(%)
0.00	0.00		0.00	0.00	0.00	0.00
0.08	68.12	60.61	71.91	64.00	76.12	
0.17	62.90	49.72			66.57	48.56
0.25	38.55	48.85	72.64	59.57	66.29	43.17
0.50	68.41	39.02	69.73	51.42	58.15	29.43
0.75	61.45	25.27	68.13	47.47	55.34	26.62
1.00	65.22	26.22	67.31	46.11	54.78	22.34
1.50	65.51	25.58	61.80	41.91	56.46	22.68
2.00	61.74	21.54	58.84	39.66	64.04	
3.00	53.91	11.84	62.23	33.18	50.59	21.31
4.00	45.51	11.63			54.49	18.40
6.00	24.68	4.70			50.00	20.72

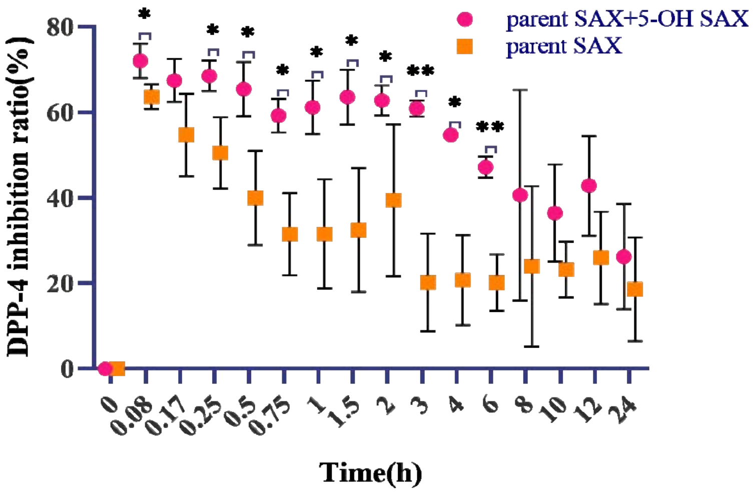

Fig.S14 A scatter plot of observed total DPP-4 inhibition ratios and parent SAX DPP-4 inhibition ratios
